# The In Vitro Stability of Circulating Tumour DNA

**DOI:** 10.1371/journal.pone.0168153

**Published:** 2016-12-13

**Authors:** Emanuela Henao Diaz, Jeffrey Yachnin, Henrik Grönberg, Johan Lindberg

**Affiliations:** 1 Department of Medical Epidemiology and Biostatistics, Science for Life Laboratory, Karolinska Institutet, Stockholm, Sweden; 2 Department of Oncology-Pathology, Karolinska Institutet and University Hospital, Stockholm, Sweden; 3 Department of Medical Epidemiology and Biostatistics, Karolinska Institutet, Stockholm, Sweden; The Ohio State University, UNITED STATES

## Abstract

**Objective:**

DNA from apoptotic cancer cells, present in the circulation, has the potential to facilitate genomic profiling and disease monitoring. However, only low fractions of total cell-free DNA originates from cancer cells, limiting the applicability of circulating tumour DNA (ctDNA). Optimal sample processing is consequently of uttermost importance. Therefore, we evaluated the in vitro stability of ctDNA.

**Experimental design:**

Blood was collected in 10 ml EDTA or Streck tubes. Three conditions (EDTA and Streck tubes in room temperature, EDTA tubes at five degrees) and four time points (plasma harvested from blood aliquots of each 10 ml tube in a time series up to 24 h) were investigated. Each condition was evaluated in five metastatic prostate cancer patients. Subsequently, three additional patients were collected enabling investigation of the in vitro stability in EDTA tubes up to 48 h.

**Methods:**

The in vitro stability of ctDNA was interrogated by low-pass whole genome sequencing which allows for the identification of somatic copy-number alterations (CNAs). In silico simulations demonstrated that non-parametric testing could detect a 1% contamination by white blood cell DNA. Mutational profiling was performed by targeted, in-solution based hybridization capture and subsequent sequencing. The allelic fraction of individual mutations was used as an estimate of the in vitro stability.

**Results:**

Somatic CNAs were detected in all patients. Surprisingly, the ctDNA levels at zero hours were not significantly different to 24 or 48 hour in vitro incubation in any investigated condition. Subsequently, mutational profiling corroborated the conclusions from the CNA analysis.

**Conclusions:**

The stability of ctDNA simplifies logistics without the requirement of immediate processing or applying fixatives to prevent white blood cell lysis.

## Introduction

Cell-free DNA (cfDNA) is present in the circulation of healthy individuals. Apoptotic cells are the most likely source as the size distribution of cfDNA corresponds to DNA bound to nucleosomes [1]. Cell-free DNA is rapidly cleared through the kidneys and possibly also degraded through enzymatic processes making it an informative biomarker for monitoring physiological states with high levels of apoptosis [2]. The most successful examples to date are non-invasive prenatal screening [3] and cancer [4] where the presence of fetal genotypes or somatic changes can be separated from germline variation. Recent work, demonstrating that cfDNA preserves tissue specific nucleosome footprints, may broaden the applicability to conditions where genotype is non-informative [5]. Circulating tumour DNA (ctDNA) has been successfully applied for monitoring of disease progression [6–8] and may replace the need of tissue-based profiles [9]. Optimal collection is a prerequisite, as contaminating white blood cell (WBC) DNA and enzymatic degradation has the potential to obscure the true ctDNA levels. Fixatives have therefore been evaluated to minimize contamination of WBC DNA for both prenatal screening [10,11] and cancer [12,13] with the drawback of preventing e.g. the establishment of patient-derived tumour models requiring live cells [14]. Recently, Kang and colleagues used mutational screening by droplet digital PCR to investigate the stability of ctDNA in multiple tube types and temperatures [15]. Four out of six patients harboured detectable low-frequency mutations in the cfDNA. Collectively, the data support the stability in EDTA tubes up to 6 h. However, there was large variability between individual time points and they did not investigate stability at 24 h. If stable at 24 h, samples could be collected in the afternoon and processed simultaneously the next day. A previous study, applied sequencing to investigate the in vitro stability of circulating cell-free fetal DNA for prenatal testing purposes. The authors concluded that the fraction of circulating cell-free fetal DNA was stable up to 48 h in EDTA tubes [16]. The extent to which in vitro contamination of WBC DNA or enzymatic processes prevent accurate detection of somatic variation in a clinical relevant time frame has, to the best of our knowledge, not been thoroughly evaluated using sequencing based assays. Therefore, before initiating a prospective collection of liquid biopsies with the goal to determine the clinical utility of ctDNA for prostate cancer, we evaluated the in vitro stability of ctDNA.

## Material and Methods

### Patients

This study was approved by the regional ethical vetting board in Stockholm (register number 2009/1357-32 and amendment registry number 2014/1564-32). Nine metastatic prostate cancer patients treated at the Karolinska University Hospital, assessed to have high tumour burden and progressive disease, were invited to participate in the study. Written informed consent was obtained from all participants.

### Sample processing

Blood was drawn into 10 ml EDTA or Streck Cell-Free DNA BCT tubes and subsequently aliquoted into standard 2 ml microcentrifuge tubes for incubation, either in room temperature or cold storage (5°C). Plasma was harvested by first spinning at 10 min in 1100 RCF. The plasma fraction was carefully transferred to another microcentrifuge tube and spun for another 10 min in 1600 RCF. The plasma was transferred to yet another microcentrifuge tube and stored in -80°C. The QIAamp Circulating Nucleic Acid Kit (Qiagen) was used to extract cell-free DNA from plasma. Library preparation was done using the ThruPLEX DNA-seq kit (Rubicon Genomics). The EZ SeqCap kit (Roche Nimblegen) was used to perform targeted sequencing for mutation detection as previously described [17]. One sequencing library of each patient (0 h in vitro incubation) had already been profiled for mutation detection in a parallel project. The panel (1.3 Mb) contains 305 genes of interest for prostate cancer, identified through extensive literature review. Paired germline DNA was also sequenced for each patient. Mutations were identified in six out of nine patients that could be used to evaluate the in vitro stability of circulating tumor DNA (ctDNA). A smaller custom design panel (160 kb) was applied to capture the same ThruPLEX libraries, used for exploration of copy-number alterations (CNAs), in the in vitro time series if there was any library left. Sequencing was performed on the Illumina Hiseq 2500 in rapid mode.

### Data analysis

Alignment to the genome was performed using BWA [18] and PCR duplicates were removed with the software suit Picard [19]. QDNAseq was applied to identify CNAs [20]. Due to small segmentation variation, the segments of one sequencing library at time-point zero were used for each patient. Each sequencing library was processed independently using QDNAseq. The median of the normalized, outlier smoothed CNA ratios was determined for each segment and sequencing library, which was subsequently used to evaluate the in vitro stability of ctDNA. To facilitate interpretation, analysis was subsequently performed on a log2 scale. Mutations were identified by applying VarDict [21] to the zero-hour plasma sample. Paired germline DNA was used to separate germline from somatic variation. Subsequently, samtools [22] was used to create pileups at the mutated positions to assess the allelic fractions across the time series. All analyses were performed in R, using built-in statistical packages for linear regression modelling, parametric and non-parametric testing [23].

## Results

**Fig 1:**
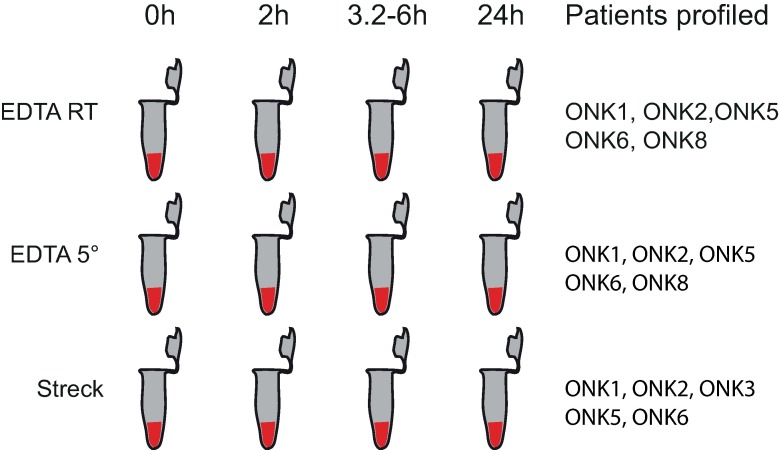
Experimental design. Blood was drawn into 10 ml EDTA and Streck Cell-Free DNA BCT tubes and subsequently aliquoted into standard 2 ml microcentrifuge tubes for incubation, either in room temperature or cold storage (5°C). Plasma was harvested at multiple time points (S1 Table) and DNA was extracted in batch before sequencing. Five biological replicates were profiled for each condition (Patients Profiled).

Blood was drawn from metastatic prostate cancer patients with high tumour burden to evaluate the in vitro stability of circulating tumour DNA (ctDNA). Three conditions (blood tube type and temperature) and four time points (up to 24 h) were investigated (Fig 1). Each condition was evaluated in five separate metastatic prostate cancer patients. As a comparator to the EDTA tubes, Streck Cell-Free DNA BCT tubes were used, which contain a fixative to stabilize white blood cells (WBC) [13].

**Fig 2:**
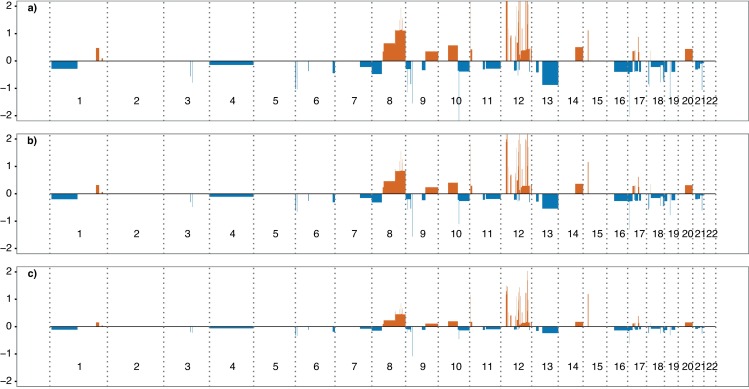
In silico dilution of cell-free DNA. **a**) Somatic copy-number alterations detected in the cell-free DNA of ONK6. **b**) As a) but the cell-free DNA was diluted to 66% by germline DNA sequencing data. **c)** As b) but diluted to 33%. Y-axis; log2 of the segmented copy-number alteration ratios. X-axis; autosomal chromosomes in order. Red colour bars; amplifications. Blue colour bars; deletions.

**Fig 3:**
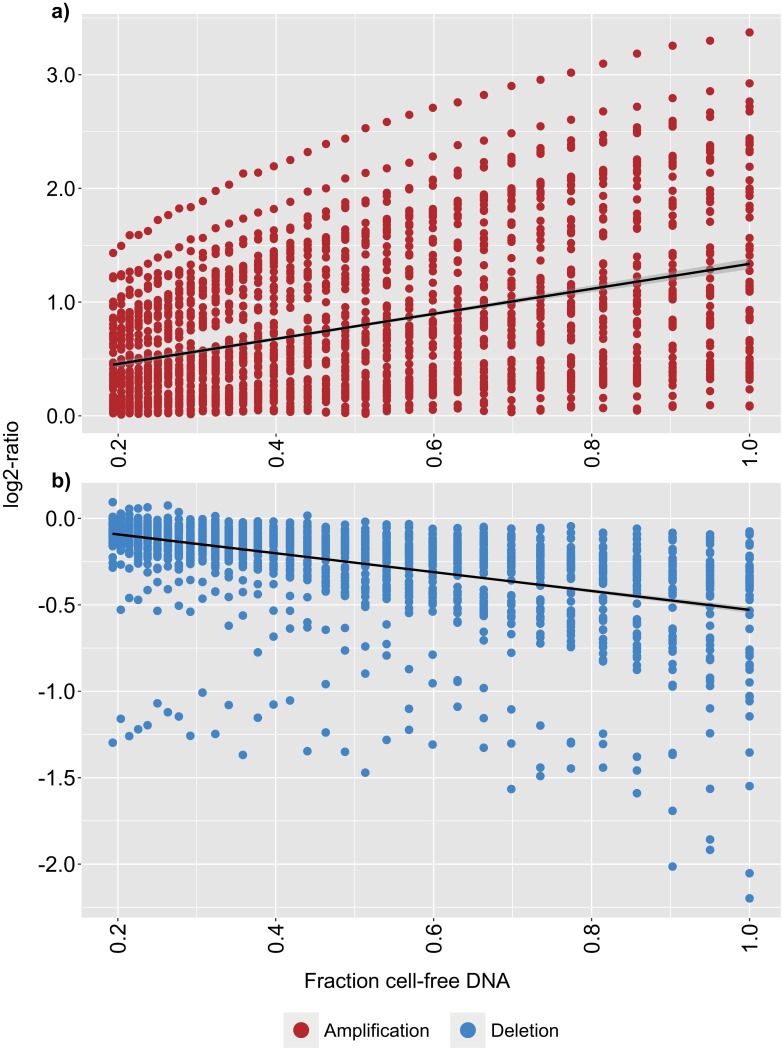
Linear regression of in silico diluted cell-free DNA. The cell-free DNA sequencing data of ONK6 was diluted with germline DNA sequencing data. Amplifications **a**) demonstrate a decrease in log2-ratio as the fraction of cell-free DNA data decreases. **b**) as a) but for deletions. The linear regression beta-coefficient was significant for both amplifications and deletions (<2e-16). Y-axis; log2 of the segmented copy-number alteration ratios. X-axis; the fraction of cell-free DNA- relatively spiked-in germline sequencing data, ranging from 19.3 to 100%.

Cell-free DNA (cfDNA) was extracted from 60 plasma samples and library prep was performed before sequencing, yielding on average 7.3 million read fragments per sample (S1 Table). Somatic copy-number alterations (CNAs) were detected in the cfDNA of all patients. To validate the sensitivity of detecting a decrease in ctDNA fraction using low-pass whole genome sequencing, WBC DNA was used to dilute the cfDNA sequencing data in silico. We assumed a five percent degradation of cfDNA per iteration with simultaneous replacement by contaminating WBC DNA. The simulation was performed on ONK6, who carried CNAs commonly detected in prostate cancer (amplification of 8q, loss of 8p, 13q, 16q, 18) [24]. The dilution of cfDNA with germline DNA resulted in evident truncation of the log2-ratios (Fig 2). As the kinetics of cfDNA stability in vitro is not known, linear regression was performed on the simulated dilution series comparing the log2-ratios of the amplifications and deletions to the fraction of cfDNA, which yielded a significant beta-coefficient associated with the reduction in ctDNA fraction (Fig 3). Additionally, to enable detection of decreasing ctDNA levels without knowledge of in vitro kinetics, a non-parametric Wilcoxon signed rank test was applied on the log2 ratios. An in silico dilution of one- and two percent cfDNA with WBC DNA was found to be highly significant (p-values: 4.5e-9, 1.3e-11 for amplifications and 6.1e-4, 2.6e-6 for deletions tested separately). The same rationale was applied to ONK3 and ONK8 with congruent results (data not shown).

**Fig 4:**
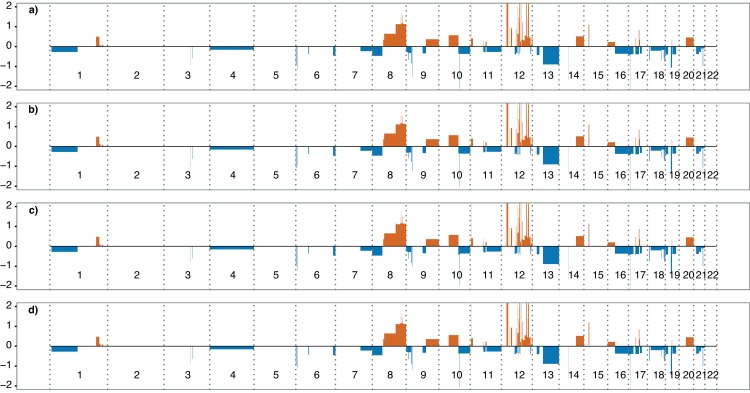
In vitro stability of ONK6 ctDNA collected in EDTA tubes and stored in room temperature. Somatic copy-number alterations detected in the cell-free DNA of ONK6. **a**) Plasma harvested immediately. **b**) Plasma harvested at 2 h. **c**) Plasma harvested at 4 h. **d**) Plasma harvested at 24 h. Y-axis; log2 of the segmented copy-number alteration ratios. X-axis; autosomal chromosomes in order. Red colour bars; amplifications. Blue colour bars; deletions.

**Fig 5:**
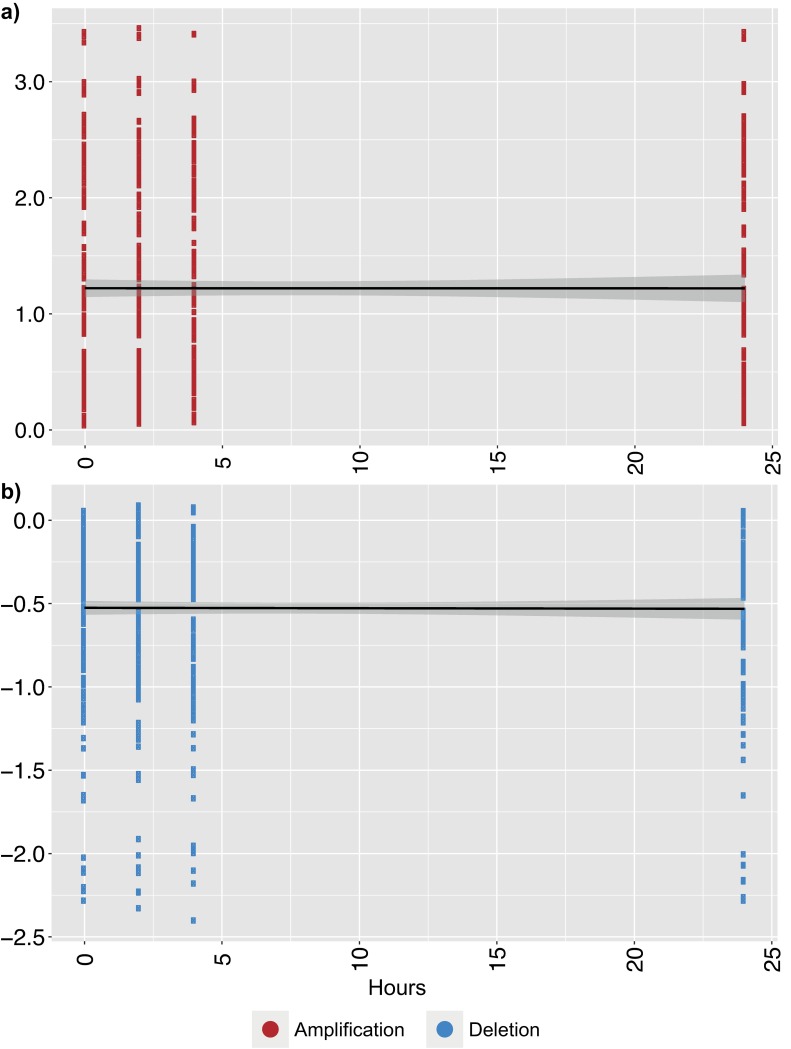
Linear regression of the in vitro stability of ONK6 ctDNA collected in EDTA tubes and stored in room temperature. Amplifications **a)** demonstrate no decrease in log2-ratio over time. **b)** as a) but for deletions. The linear regression beta-coefficient was not significantly different from 0 for either amplifications or deletions (S3 Table).

Next, the blood samples of ONK6, where plasma was obtained at 2, 4 and 24 h were tested relatively to the plasma harvested at 0 h. Surprisingly, there was no significant difference in ctDNA fraction by non-parametric testing, not even at 24 h for any condition (Fig 4, S1 and S2 Figs, S2 Table). Accordingly, applying linear regression did not either reveal any reduction in signal, as the slope was not significantly different from zero (Fig 5, S3 Table). As CNAs were detected in the cfDNA of all patients, the same approach was applied to all samples, which confirmed the in vitro stability of ctDNA in plasma processed up to 24 h after venipuncture (S3–S14 Figs, S2 and S3 Tables).

To explore if ctDNA remained stable in vitro over a longer period of time, blood was drawn into EDTA tubes from three additional patients, comparing plasma purification at 0 h to 48 h, in room temperature and cold storage (9 million reads in average per sample). Even at 48 h, the signal remained constant (S15–S17 Figs, S2 and S3 Tables). The only significant associations were amplifications of ONK5 (0h vs. 24h) and deletions of ONK15 (0 h vs. 48 h). However, the deletions and amplifications of ONK5 and ONK15, respectively, were not associated with a reduction in ctDNA signal (S2 Table). Additionally, the linear regression did not provide any evidence supporting loss of ctDNA signal (S3 Table).

**Fig 6:**
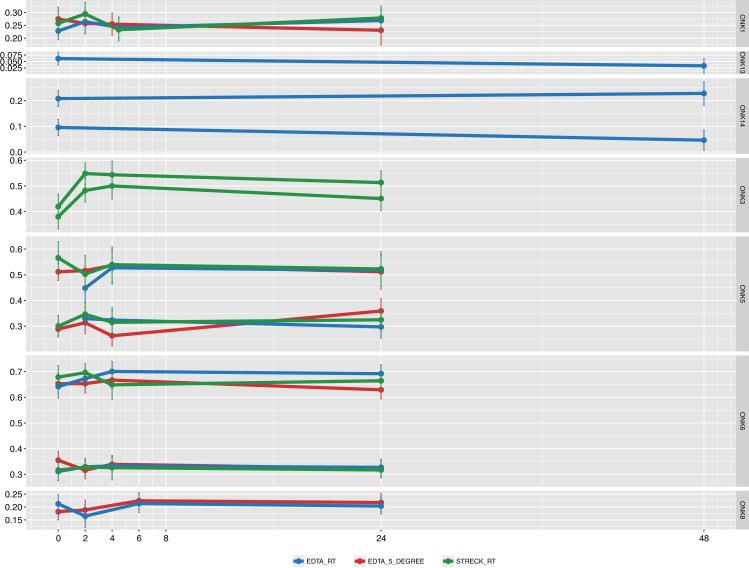
In vitro stability of ctDNA investigated by mutational profiling. Y-axis; the fraction of each mutation in the cfDNA. X-axis; the time points when plasma was harvested. The error bars display a 95% confidence interval of the estimated mutant allele fraction. Tube type and storage temperature—bottom legend. Patient ID—right panel label. Note, two informative mutations were identified for ONK3, ONK5, ONK6 and ONK14.

To verify the stability observed by analysis of genome-wide CNAs we performed mutational profiling by targeted in-solution based hybridization capture and subsequent Illumina sequencing (S4 Table). An initial screen of the plasma samples that were harvested at 0 h identified mutations in six out of nine patients (S5 Table). For these patients, mutational profiling was performed on the 52 samples where library material remained after low-pass whole genome sequencing (Fig 6, average coverage: 617). Only one library failed capture (ONK5, EDTA tube, 0h incubation). Normal approximation of the binomial distribution was applied to estimate the standard error of the mutational fractions and to investigate the in vitro stability. A one-sided test was performed to determine if the proportion of reads in the time series was greater than the last time point (24h or 48 h) for each patient, mutation and condition (S6 Table). This corroborated the stability observed by CNA analysis.

## Discussion

There are multiple emerging avenues for treating metastatic prostate cancer. A recent study demonstrated DNA-repair deficiency in approximately 20% of castrate resistant patients [25], for which PARP inhibitors demonstrate promising effects [26]. Additionally, immune checkpoint therapy is highly likely to benefit hypermutated prostate cancers as recently reported for mismatch-repair deficient tumours of different origins [27]. The common denominator of these novel therapies is the need of molecular profiling, restricting treatment to patients with tumours harbouring the somatic events associated with response to treatment. This will not only reduce cost, but also avoid unnecessary side effects. As the majority of prostate cancers metastasize to the bone, with low success rate in obtaining sufficient material for genomic profiling, liquid biopsies is an appealing alternative. Therefore, we have initiated a prospective study at the Karolinska Hospital, with the goal to evaluate the feasibility of replacing tumour tissue based profiles with liquid biopsies. During peaks days, up to five patients will be included, putting high demands on personnel availability if sample quality is dependent on immediate processing. As circulating tumour cells experiments will be performed on cherry-picked samples, we wanted to avoid fixatives for simplicity when distributing blood tubes. Surprisingly, there were no solid data in the literature on the in vitro stability of ctDNA using sequencing based assays. Therefore, we set up a time-series harvesting blood in conventional EDTA tubes, both in cold storage and room temperature up to 24 h. As control we used the Streck Cell-Free DNA BCT tubes, containing a fixative preventing WBC lysis [13]. Circulating tumour DNA was profiled using low-pass whole genome sequencing [28], allowing assessment of multiple somatic events to detect any effects due to in vitro incubation. Surprisingly, the result from five biological replicates of each condition (blood tube and temperature) consistently did not demonstrate any reduction in ctDNA levels during 24 h. Therefore, we collected blood from three additional patients, allowing us to compare immediate harvesting of plasma with 48 h EDTA tube storage in room temperature and cold storage. Even here, the ctDNA fraction was stable. How does these data reconcile previous literature? It is evident that blood kept in EDTA tubes for 24 h or longer will contain contaminating WBC DNA [10,12]. However, as cfDNA fragments are short (<200 nt), fragmentation is not applied when preparing DNA harvested from plasma for sequencing. If contaminating WBC DNA is significantly larger than cfDNA and remains intact, it will be outcompeted by the shorter cfDNA during PCR and would not be able to form clusters on the Illumina flow-cell with the same efficiency. Accordingly, the 48 h plasma sample of ONK14 contained contaminating long fragments, which were undetectable when analysing the size distribution of the sequencing data obtained (S18 Fig). Similarly conclusions can be extrapolated from previous studies, investigating the in-vitro stability of cfDNA for prenatal testing. Barret and colleagues concluded that the total concentration of DNA present in plasma was increased at 24 h due to contamination of WBC DNA. However, the WBC DNA was not fragmented to the same size-range as cfDNA and the concentration of cfDNA remained constant [29]. Additionally, a subsequent study applying sequencing to determine the fetal fraction in cfDNA of pregnant women could not detect any decrease up to 48 h [16]. To further confirm our conclusions from the CNA analysis, we performed mutational profiling in six of the nine patients investigated here, where mutations could be detected. This analysis was in concordance with the CNA analysis, demonstrating no significant decrease in ctDNA fraction in any of the investigated conditions.

In summary, these data demonstrate that ctDNA is stable in plasma for at least 24 h, possibly up to 48 h, although only three samples were profiled at this time point. This provides the possibility to perform high-quality ctDNA profiling either by performing size selection or by choosing methodology, such as Illumina sequencing, where only short fragments are accessible. For single-centre studies obtaining blood samples from multiple patients during one day, all plasma may be harvested simultaneously in the late afternoon without compromising quality. However, we did not test the stability in a multi-centre context, e.g. by shipping EDTA tube using couriers. If variable temperatures and rough handling of samples would lead to fragmentation of WBC DNA and an attenuation of the ctDNA signal remains to be tested. Nevertheless, this study brings legitimacy to previous ctDNA-based literature, where plasma preparation is typically performed at a single centre within a variable timeframe during one working day.

## Supporting Information

S1 Table Sequencing metrics and sample information. Provides an overview of the sequencing data generated; the time points for harvesting plasma; storage temperature and which tubes used. Columns in order; ID–the ID used for each patient; TOTAL_MAPPED_READS–the number of mapped reads obtained for each sequencing library; TYPE–the purpose of each sequencing library; DNA_SOURCE–the DNA source of each sequencing library; TIMEPOINT–for the in vitro time series, the time point of harvesting plasma; STORAGE–for the in vitro time series, if room temperature or cold storage was applied; TUBE–blood collection tube type.
(XLSX)

S2 Table Summarizes the Wilcoxon signed rank test comparing the log2 ratios between immediately harvested cfDNA to subsequent time points. Columns in order; ID–the ID used for each patient; TUBE–blood collection tube type; STORAGE–for the in vitro time series, if room temperature or cold storage was applied; TIMEPOINT_1 –the first time point tested; TIMEPOINT_2 –the second time point tested; PVAL–the p-value of the statistical test; TYPE–if amplifications or deletions were used to test for differences in log2 ratios; BONFERRONI–Bonferroni correction of the p-values.
(XLSX)

S3 Table Linear regression of the log2 ratios for the in vitro time series. Columns in order; ID–the ID used for each patient; TUBE–blood collection tube type; STORAGE–for the in vitro time series, if room temperature or cold storage was applied; ALPHA–the intercept of the linear regression; BETA–the slope of the linear regression; ALPHA_PVALUE–the probability of alpha = 0; BETA_PVALUE–the probability of beta = 0; TYPE–if amplifications or deletions were used for linear regression; BONFERRONI_ALPHA—Bonferroni correction of the p-values for the intercept of the linear regression; BONFERRONI_BETA–Bonferroni correction of the p-values for the regression coefficient beta.
(XLSX)

S4 Table Sequencing metrics for targeted sequencing. Provides an overview of the sequencing data generated; the time points for harvesting plasma; storage temperature and which tubes used. Columns in order; ID–the ID used for each patient; PANEL_SIZE (MB)–the size of the genome that was captured and sequenced; MEAN_TARGET_COVERAGE–the mean target coverage in the panel sequenced; FRACTION_DUPLICATION–the fraction of sequencing duplicates in the sequencing library; TOTAL_MAPPED_READS–the number of unique read pairs obtained for each sequencing library that mapped to the human genome; TYPE–the purpose of each sequencing library; DNA_SOURCE–the DNA source of each sequencing library; TIMEPOINT–for the in vitro time series, the time point of harvesting plasma; STORAGE–for the in vitro time series, if room temperature or cold storage was applied; TUBE–blood collection tube type.
(XLSX)

S5 Table Mutations detected by targeted sequencing. Columns in order; CHROM–the chromosome harboring the mutation; POS–the position of the mutation; REF–reference allele; ALT–the mutated allele; MUTATION_EFFECT–the consequence of the identified mutation; AMINO_ACID_CHANGE–the change to the amino acid sequence as a consequence of the mutation; GENE_NAME–the name of the affected gene; ID–the ID used for each patient; TIMEPOINT–for the in vitro time series, the time point of harvesting plasma; STORAGE–for the in vitro time series, if room temperature or cold storage was applied; TUBE–blood collection tube type.
(XLSX)

S6 Table Summarizes statistical testing for a decrease in allelic fraction in the time series. CHROM–the chromosome harboring the mutation; POS–the position of the mutation; REF–reference allele; ALT–the mutated allele; MUTATION_EFFECT–the consequence of the identified mutation; GENE_NAME–the name of the affected gene; NBR_REF_READS–the number of reference alleles in this position; NBR_VARIANT_READS–the number of mutated alleles in this position; NBR_BACKGROUND_READS–the number of non reference/mutant reads at this position; TOT_COUNT–total read depth at this position; ALLELIC_FRACTION–the fraction of mutated reads at this position; ID–the ID used for each patient; STORAGE–for the in vitro time series, if room temperature or cold storage was applied; TUBE–blood collection tube type; TIMEPOINT–for the in vitro time series, the time point of harvesting plasma; PVAL–the p-value of the statistical test. A one-sided test was performed to test if the proportion of reads in the time series was greater than the last time point (24h or 48 h); BONFERRONI–Bonferroni correction of the p-values.
(XLSX)

S1 Fig In vitro stability of ONK6 ctDNA collected in EDTA tubes and stored at 5°C. Somatic copy-number alterations detected in the cell-free DNA of ONK6. a) Plasma harvested immediately. b) Plasma harvested at 2 h. c) Plasma harvested at 4 h. d) Plasma harvested at 24 h. Y-axis; log2 of the segmented copy-number alteration ratios. X-axis; autosomal chromosomes in order. Red colour bars; amplifications. Blue colour bars; deletions.
(PDF)

S2 Fig In vitro stability of ONK6 ctDNA collected in Streck Cell-Free DNA BCT tubes and stored in room temperature. Somatic copy-number alterations detected in the cell-free DNA of ONK6. a) Plasma harvested immediately. b) Plasma harvested at 2 h. c) Plasma harvested at 4 h. d) Plasma harvested at 24 h. Y-axis; log2 of the segmented copy-number alteration ratios. X-axis; autosomal chromosomes in order. Red colour bars; amplifications. Blue colour bars; deletions.
(PDF)

S3 Fig In vitro stability of ONK1 ctDNA collected in EDTA tubes and stored in room temperature. Somatic copy-number alterations detected in the cell-free DNA of ONK1. a) Plasma harvested immediately. b) Plasma harvested at 2 h. c) Plasma harvested at 4.5 h. d) Plasma harvested at 24 h. Y-axis; log2 of the segmented copy-number alteration ratios. X-axis; autosomal chromosomes in order. Red colour bars; amplifications. Blue colour bars; deletions.
(PDF)

S4 Fig In vitro stability of ONK1 ctDNA collected in EDTA tubes and stored at 5°C. Somatic copy-number alterations detected in the cell-free DNA of ONK1. a) Plasma harvested immediately. b) Plasma harvested at 2 h. c) Plasma harvested at 4.5 h. d) Plasma harvested at 24 h. Y-axis; log2 of the segmented copy-number alteration ratios. X-axis; autosomal chromosomes in order. Red colour bars; amplifications. Blue colour bars; deletions.
(PDF)

S5 Fig In vitro stability of ONK1 ctDNA collected in Streck Cell-Free DNA BCT tubes and stored in room temperature. Somatic copy-number alterations detected in the cell-free DNA of ONK1. a) Plasma harvested immediately. b) Plasma harvested at 2 h. c) Plasma harvested at 4.5 h. d) Plasma harvested at 24 h. Y-axis; log2 of the segmented copy-number alteration ratios. X-axis; autosomal chromosomes in order. Red colour bars; amplifications. Blue colour bars; deletions.
(PDF)

S6 Fig In vitro stability of ONK2 ctDNA collected in EDTA tubes and stored in room temperature. Somatic copy-number alterations detected in the cell-free DNA of ONK2. a) Plasma harvested immediately. b) Plasma harvested at 2 h. c) Plasma harvested at 3.2 h. d) Plasma harvested at 24 h. Y-axis; log2 of the segmented copy-number alteration ratios. X-axis; autosomal chromosomes in order. Red colour bars; amplifications. Blue colour bars; deletions.
(PDF)

S7 Fig In vitro stability of ONK2 ctDNA collected in EDTA tubes and stored at 5°C. Somatic copy-number alterations detected in the cell-free DNA of ONK1. a) Plasma harvested immediately. b) Plasma harvested at 2 h. c) Plasma harvested at 3.8 h. d) Plasma harvested at 24 h. Y-axis; log2 of the segmented copy-number alteration ratios. X-axis; autosomal chromosomes in order. Red colour bars; amplifications. Blue colour bars; deletions.
(PDF)

S8 Fig In vitro stability of ONK2 ctDNA collected in Streck Cell-Free DNA BCT tubes and stored in room temperature. Somatic copy-number alterations detected in the cell-free DNA of ONK1. (a) Plasma harvested immediately. (b) Plasma harvested at 2 h. (c) Plasma harvested at 3.2 h. (b) Plasma harvested at 24 h. Y-axis; log2 of the segmented copy-number alteration ratios. X-axis; autosomal chromosomes in order. Red colour bars; amplifications. Blue colour bars; deletions.
(PDF)

S9 Fig In vitro stability of ONK3 ctDNA collected in Streck Cell-Free DNA BCT tubes and stored in room temperature. Somatic copy-number alterations detected in the cell-free DNA of ONK1. a) Plasma harvested immediately. b) Plasma harvested at 2 h. c) Plasma harvested at 4 h. d) Plasma harvested at 24 h. Y-axis; log2 of the segmented copy-number alteration ratios. X-axis; autosomal chromosomes in order. Red colour bars; amplifications. Blue colour bars; deletions.
(PDF)

S10 Fig In vitro stability of ONK5 ctDNA collected in EDTA tubes and stored in room temperature. Somatic copy-number alterations detected in the cell-free DNA of ONK2. a) Plasma harvested immediately. b) Plasma harvested at 2 h. c) Plasma harvested at 4 h. d) Plasma harvested at 24 h. Y-axis; log2 of the segmented copy-number alteration ratios. X-axis; autosomal chromosomes in order. Red colour bars; amplifications. Blue colour bars; deletions.
(PDF)

S11 Fig In vitro stability of ONK5 ctDNA collected in EDTA tubes and stored at 5°C. Somatic copy-number alterations detected in the cell-free DNA of ONK1. a) Plasma harvested immediately. b) Plasma harvested at 2 h. c) Plasma harvested at 4 h. d) Plasma harvested at 24 h. Y-axis; log2 of the segmented copy-number alteration ratios. X-axis; autosomal chromosomes in order. Red colour bars; amplifications. Blue colour bars; deletions.
(PDF)

S12 Fig In vitro stability of ONK5 ctDNA collected in Streck Cell-Free DNA BCT tubes and stored in room temperature. Somatic copy-number alterations detected in the cell-free DNA of ONK1. a) Plasma harvested immediately. b) Plasma harvested at 2 h. c) Plasma harvested at 4 h. d) Plasma harvested at 24 h. Y-axis; log2 of the segmented copy-number alteration ratios. X-axis; autosomal chromosomes in order. Red colour bars; amplifications. Blue colour bars; deletions.
(PDF)

S13 Fig In vitro stability of ONK8 ctDNA collected in EDTA tubes and stored in room temperature. Somatic copy-number alterations detected in the cell-free DNA of ONK2. a) Plasma harvested immediately. b) Plasma harvested at 2 h. c) Plasma harvested at 6 h. d) Plasma harvested at 24 h. Y-axis; log2 of the segmented copy-number alteration ratios. X-axis; autosomal chromosomes in order. Red colour bars; amplifications. Blue colour bars; deletions.
(PDF)

S14 Fig In vitro stability of ONK8 ctDNA collected in EDTA tubes and stored at 5°C. Somatic copy-number alterations detected in the cell-free DNA of ONK1. a) Plasma harvested immediately. b) Plasma harvested at 2 h. c) Plasma harvested at 6 h. d) Plasma harvested at 24 h. Y-axis; log2 of the segmented copy-number alteration ratios. X-axis; autosomal chromosomes in order. Red colour bars; amplifications. Blue colour bars; deletions.
(PDF)

S15 Fig In vitro stability of ONK13 ctDNA collected in EDTA tubes and stored in room temperature. Somatic copy-number alterations detected in the cell-free DNA of ONK13. a) Plasma harvested immediately. b) Plasma harvested at 48 h. Y-axis; log2 of the segmented copy-number alteration ratios. X-axis; autosomal chromosomes in order. Red colour bars; amplifications. Blue colour bars; deletions.
(PDF)

S16 Fig In vitro stability of ONK14 ctDNA collected in EDTA tubes and stored in room temperature. Somatic copy-number alterations detected in the cell-free DNA of ONK14. a) Plasma harvested immediately. b) Plasma harvested at 48 h. Y-axis; log2 of the segmented copy-number alteration ratios. X-axis; autosomal chromosomes in order. Red colour bars; amplifications. Blue colour bars; deletions.
(PDF)

S17 Fig In vitro stability of ONK15 ctDNA collected in EDTA tubes and stored at 5°C. Somatic copy-number alterations detected in the cell-free DNA of ONK14. a) Plasma harvested immediately. b) Plasma harvested at 48 h. Y-axis; log2 of the segmented copy-number alteration ratios. X-axis; autosomal chromosomes in order. Red colour bars; amplifications. Blue colour bars; deletions.
(PDF)

S18 Fig The size distributions of ONK14 cfDNA and sequencing data. a) ONK14 plasma DNA was harvested at 48 hours and analysed on the Agilent Bioanalyzer. Large fragments >350 bp is typically not detected in high-quality cfDNA. Y-axis; arbitrary fluorescence units. X-axis; DNA fragment size in base pairs. b) The size distribution of the sequencing data. The high-molecular weight DNA is not detectable. Y-axis; fraction of all reads. X-axis; DNA fragment size in base pairs estimated from the sequencing data.
(PDF)
