# Supplementary figures and images for: The In Vitro Stability of Circulating Tumour DNA
Source: PLoS One. 2016 Dec 13;11(12):e0168153. doi: 10.1371/journal.pone.0168153 (PMC5154581; doi:10.1371/journal.pone.0168153)

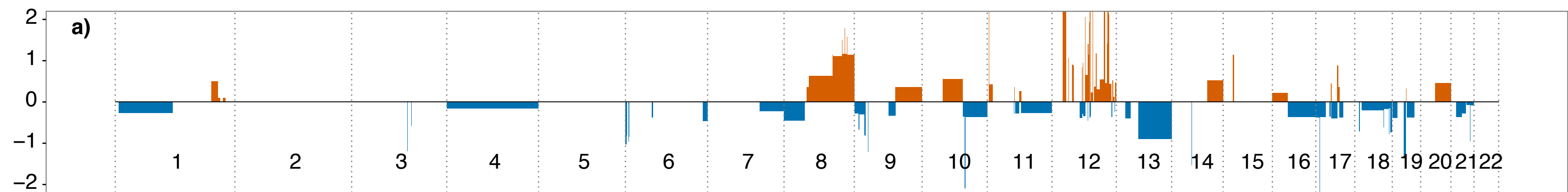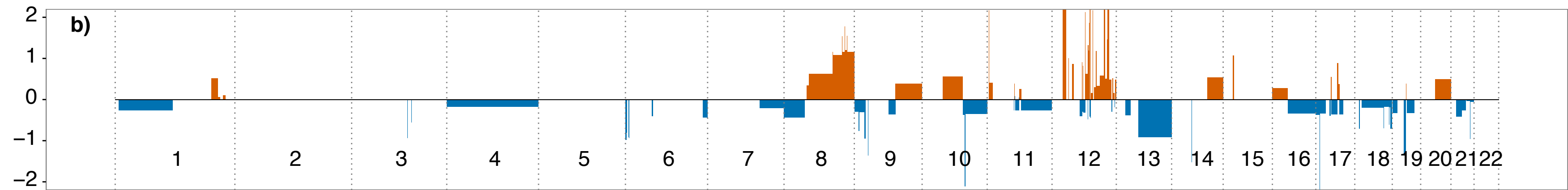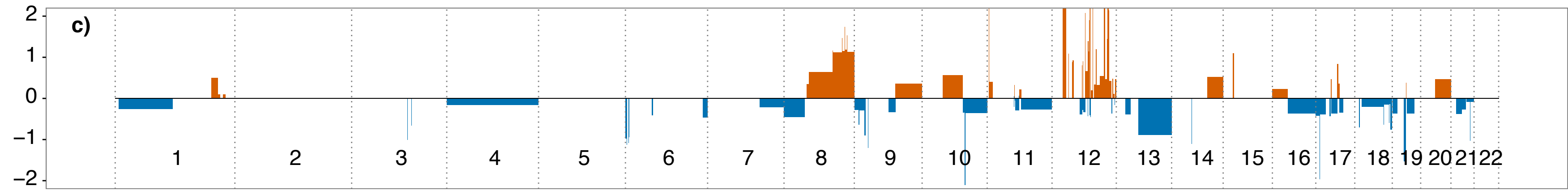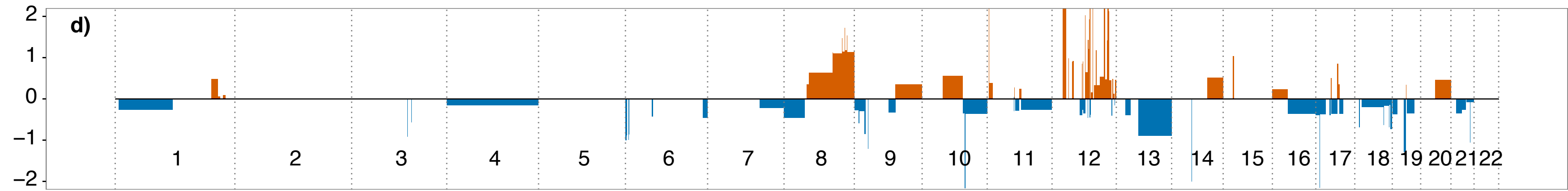

Supplement: S1 Fig — Somatic copy-number alterations detected in the cell-free DNA of ONK6. a) Plasma harvested immediately. b) Plasma harvested at 2 h. c) Plasma harvested at 4 h. d) Plasma harvested at 24 h. Y-axis; log2 of the segmented copy-number alteration ratios. X-axis; autosomal chromosomes in order. Red colour bars; amplifications. Blue colour bars; deletions. (PDF) [file pone.0168153.s007.pdf]

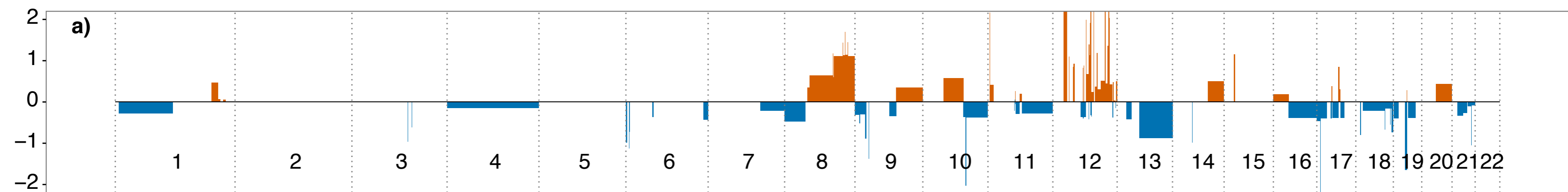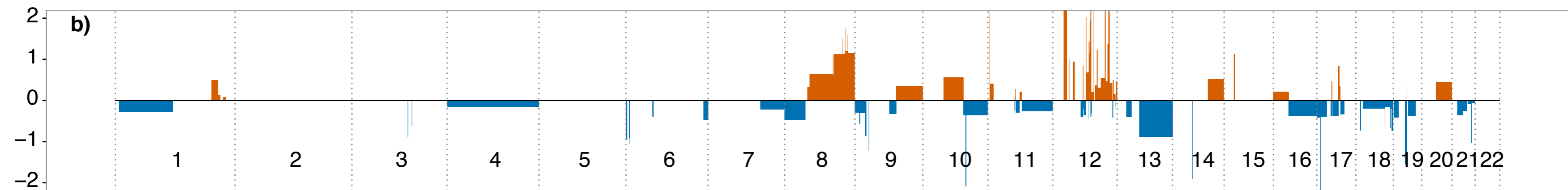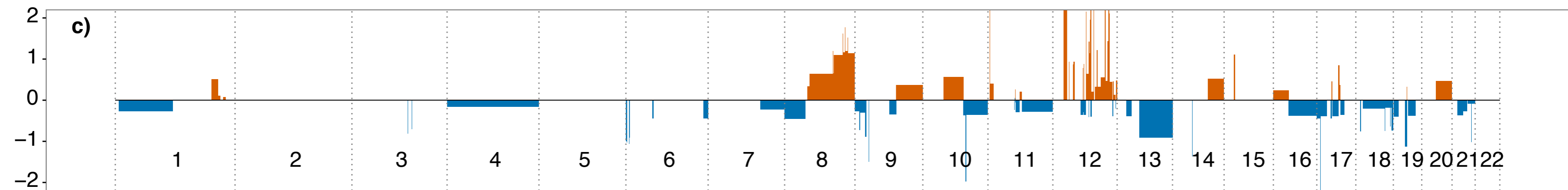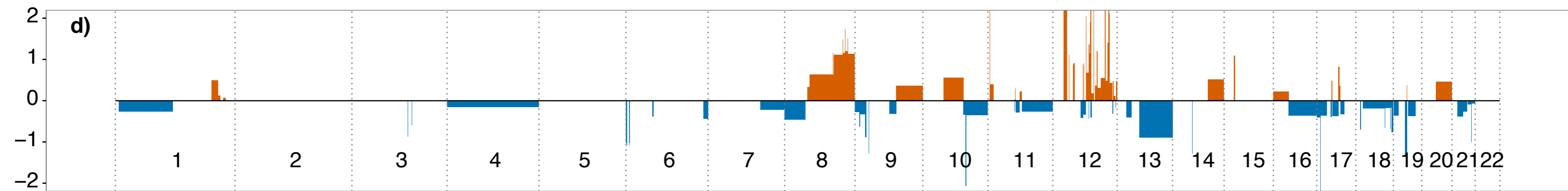

Supplement: S2 Fig — Somatic copy-number alterations detected in the cell-free DNA of ONK6. a) Plasma harvested immediately. b) Plasma harvested at 2 h. c) Plasma harvested at 4 h. d) Plasma harvested at 24 h. Y-axis; log2 of the segmented copy-number alteration ratios. X-axis; autosomal chromosomes in order. Red colour bars; amplifications. Blue colour bars; deletions. (PDF) [file pone.0168153.s008.pdf]

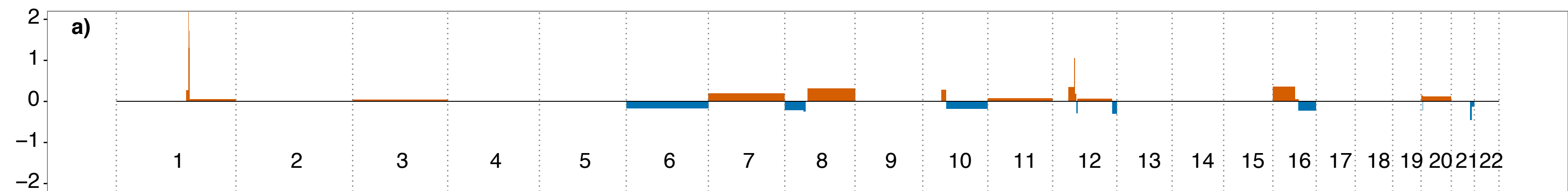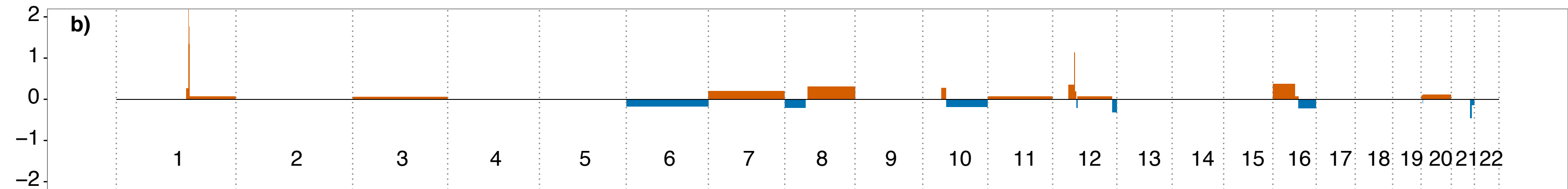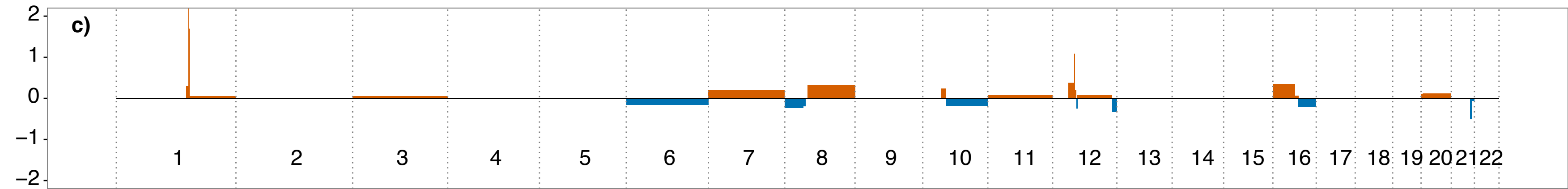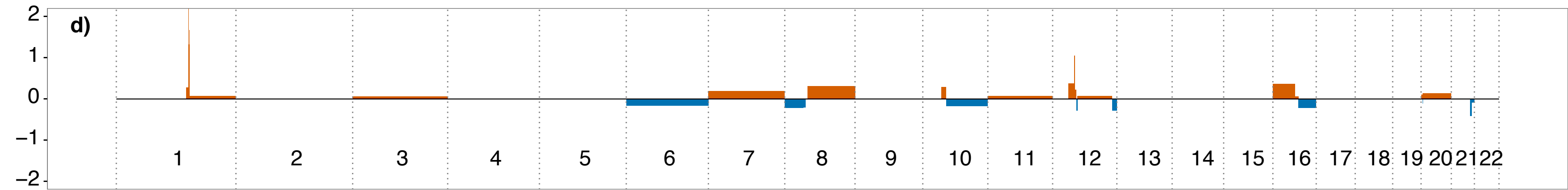

Supplement: S3 Fig — Somatic copy-number alterations detected in the cell-free DNA of ONK1. a) Plasma harvested immediately. b) Plasma harvested at 2 h. c) Plasma harvested at 4.5 h. d) Plasma harvested at 24 h. Y-axis; log2 of the segmented copy-number alteration ratios. X-axis; autosomal chromosomes in order. Red colour bars; amplifications. Blue colour bars; deletions. (PDF) [file pone.0168153.s009.pdf]

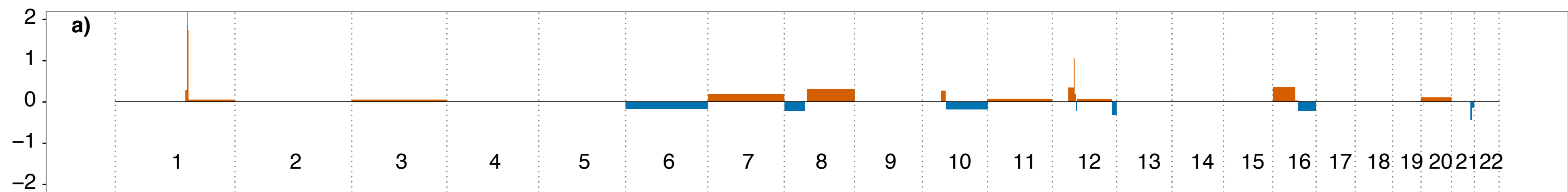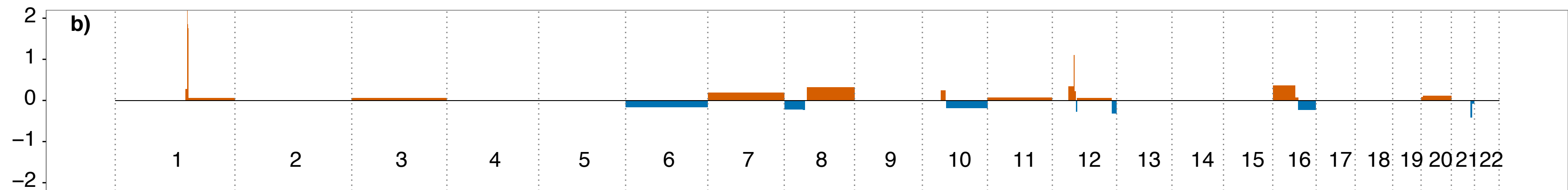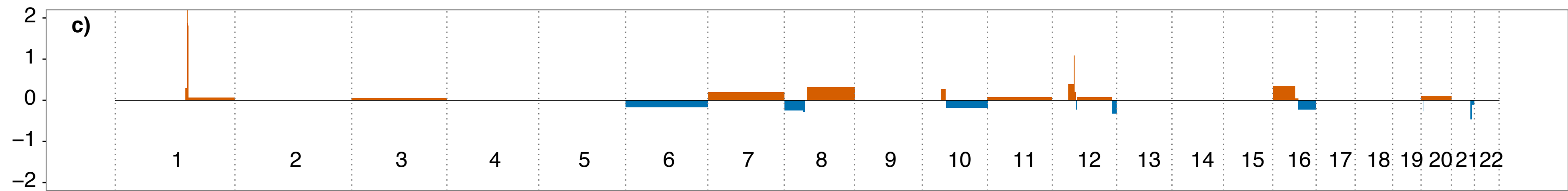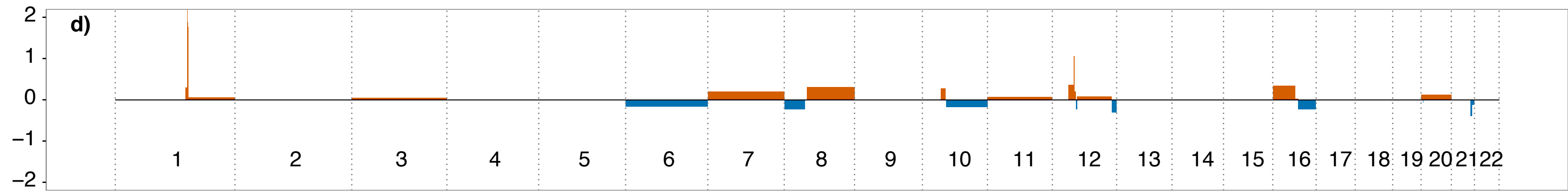

Supplement: S4 Fig — Somatic copy-number alterations detected in the cell-free DNA of ONK1. a) Plasma harvested immediately. b) Plasma harvested at 2 h. c) Plasma harvested at 4.5 h. d) Plasma harvested at 24 h. Y-axis; log2 of the segmented copy-number alteration ratios. X-axis; autosomal chromosomes in order. Red colour bars; amplifications. Blue colour bars; deletions. (PDF) [file pone.0168153.s010.pdf]

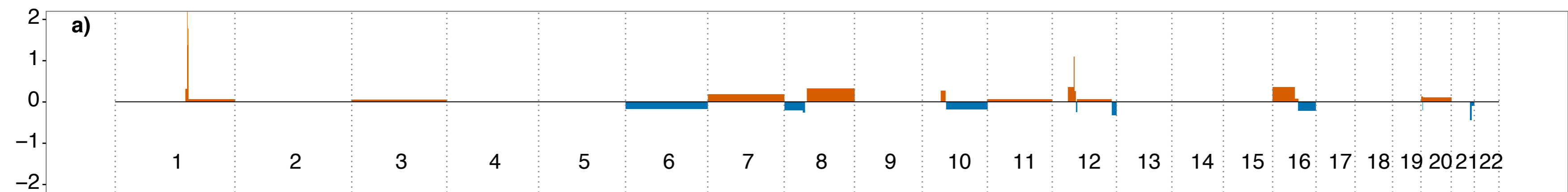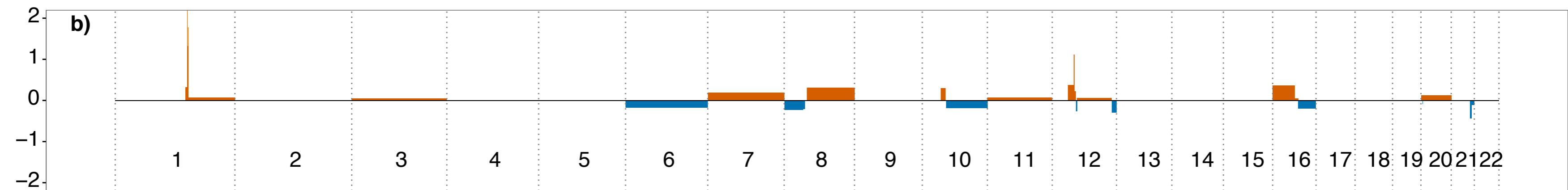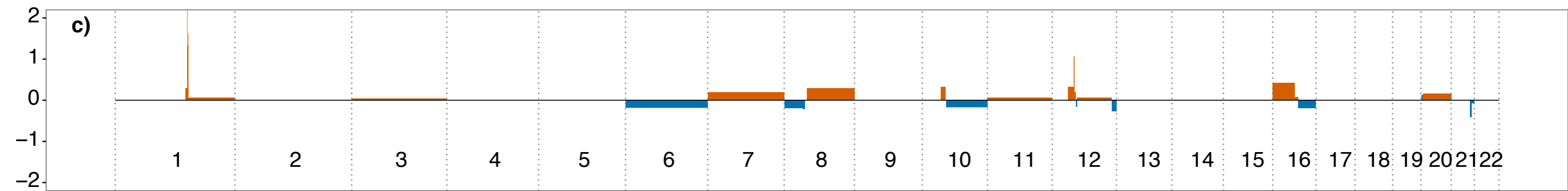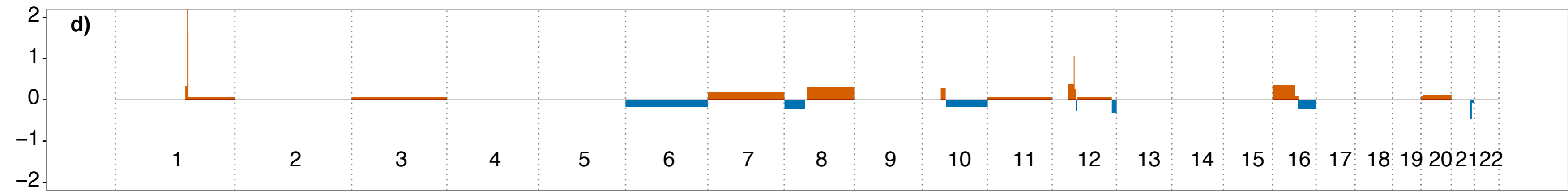

Supplement: S5 Fig — Somatic copy-number alterations detected in the cell-free DNA of ONK1. a) Plasma harvested immediately. b) Plasma harvested at 2 h. c) Plasma harvested at 4.5 h. d) Plasma harvested at 24 h. Y-axis; log2 of the segmented copy-number alteration ratios. X-axis; autosomal chromosomes in order. Red colour bars; amplifications. Blue colour bars; deletions. (PDF) [file pone.0168153.s011.pdf]

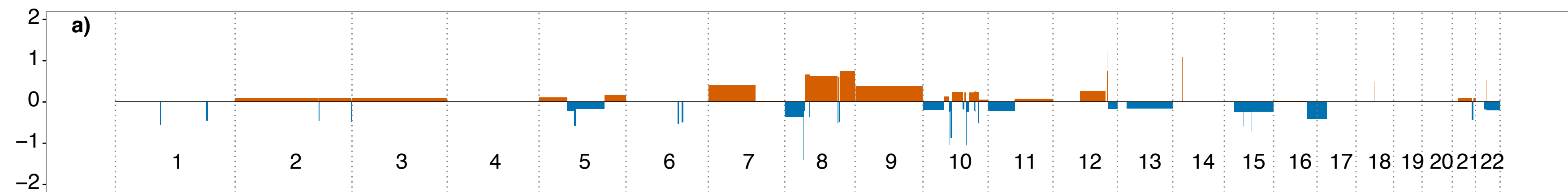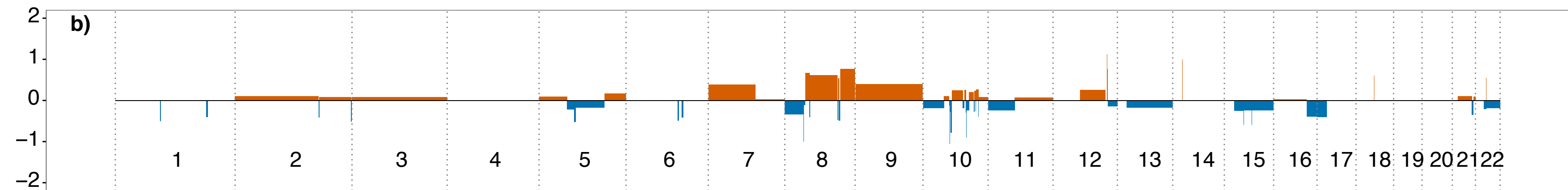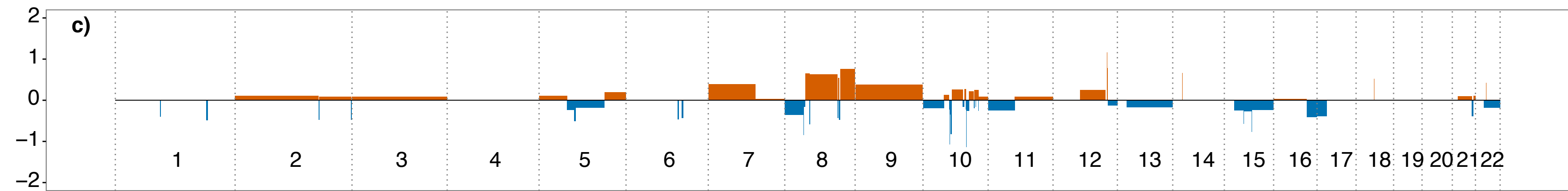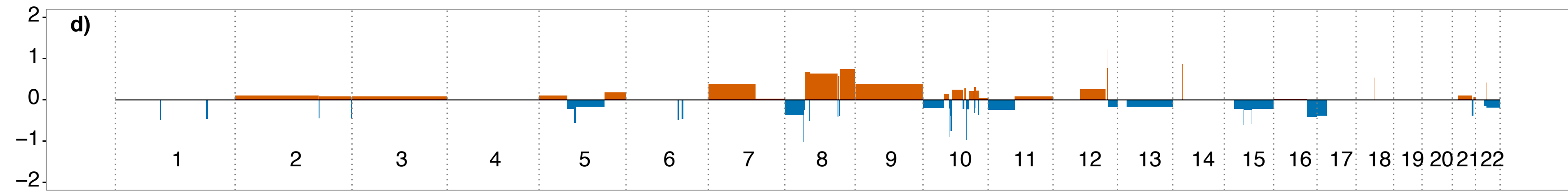

Supplement: S6 Fig — Somatic copy-number alterations detected in the cell-free DNA of ONK2. a) Plasma harvested immediately. b) Plasma harvested at 2 h. c) Plasma harvested at 3.2 h. d) Plasma harvested at 24 h. Y-axis; log2 of the segmented copy-number alteration ratios. X-axis; autosomal chromosomes in order. Red colour bars; amplifications. Blue colour bars; deletions. (PDF) [file pone.0168153.s012.pdf]

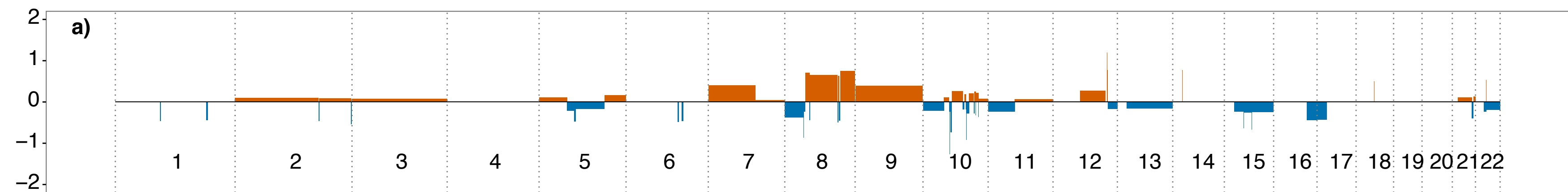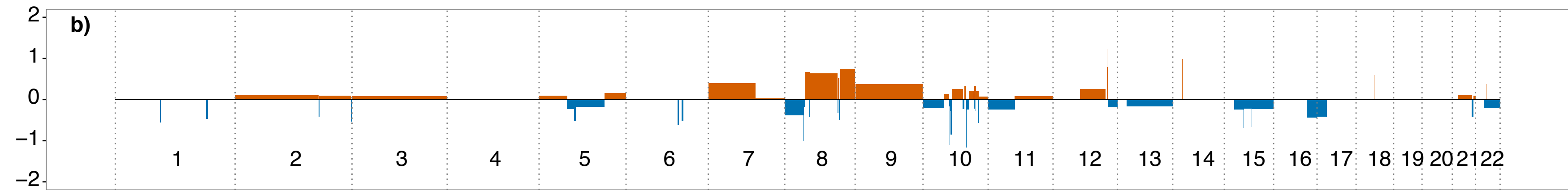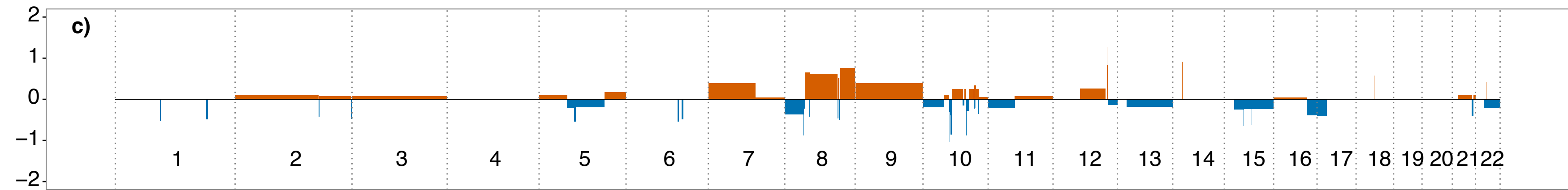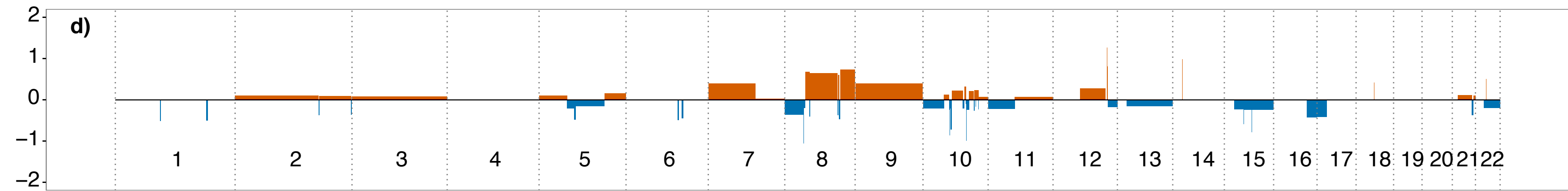

Supplement: S7 Fig — Somatic copy-number alterations detected in the cell-free DNA of ONK1. a) Plasma harvested immediately. b) Plasma harvested at 2 h. c) Plasma harvested at 3.8 h. d) Plasma harvested at 24 h. Y-axis; log2 of the segmented copy-number alteration ratios. X-axis; autosomal chromosomes in order. Red colour bars; amplifications. Blue colour bars; deletions. (PDF) [file pone.0168153.s013.pdf]

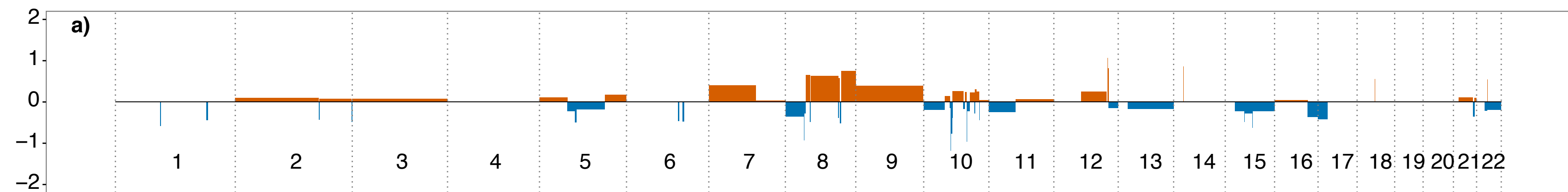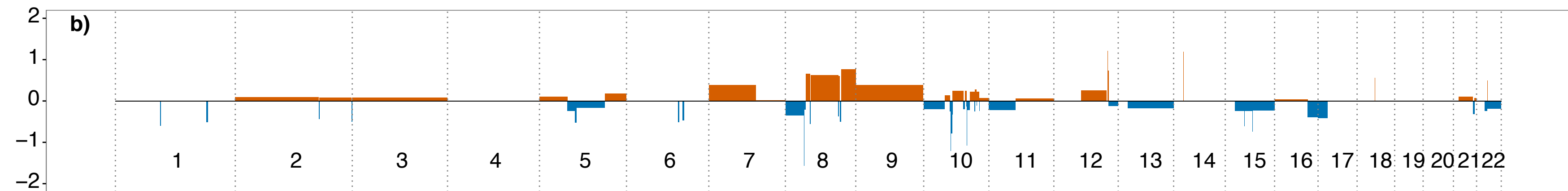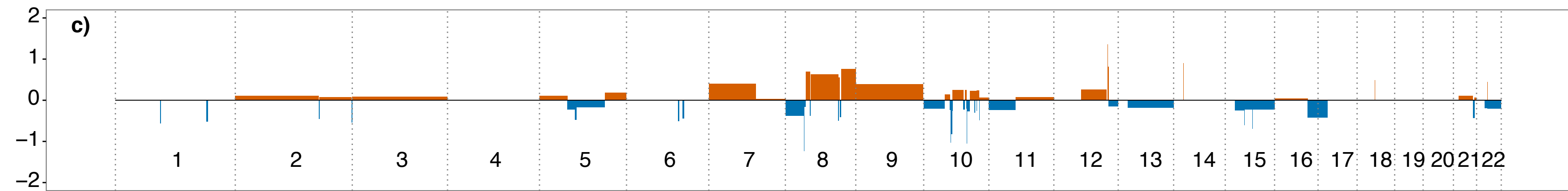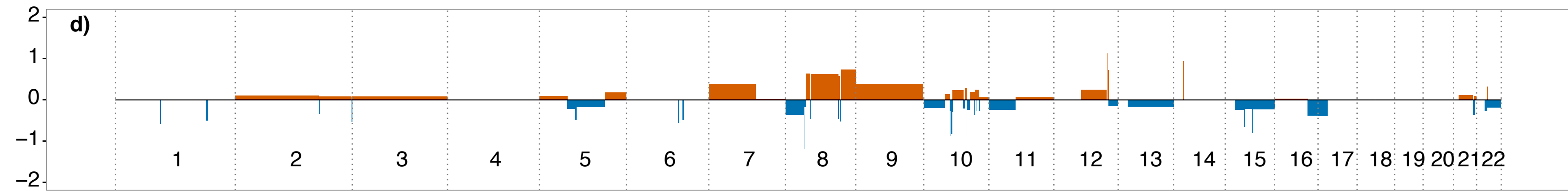

Supplement: S8 Fig — Somatic copy-number alterations detected in the cell-free DNA of ONK1. (a) Plasma harvested immediately. (b) Plasma harvested at 2 h. (c) Plasma harvested at 3.2 h. (b) Plasma harvested at 24 h. Y-axis; log2 of the segmented copy-number alteration ratios. X-axis; autosomal chromosomes in order. Red colour bars; amplifications. Blue colour bars; deletions. (PDF) [file pone.0168153.s014.pdf]

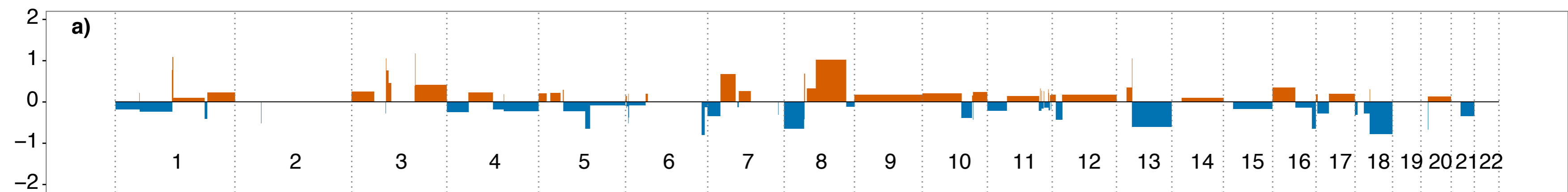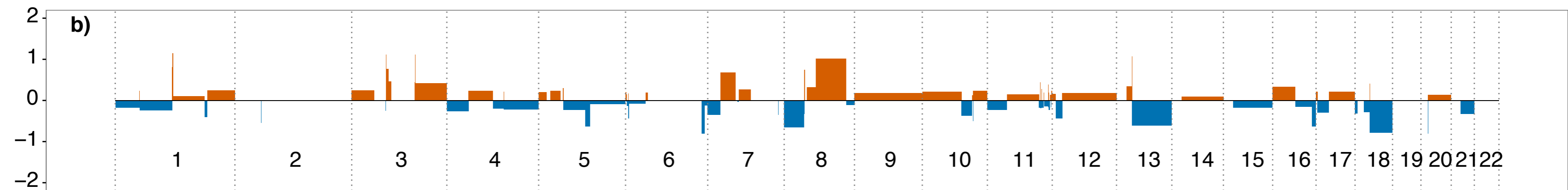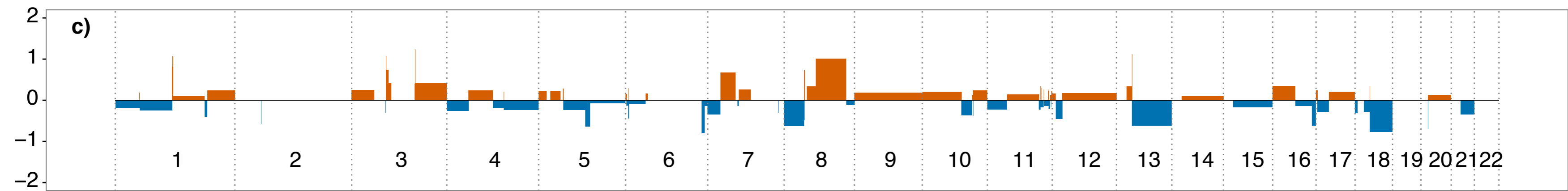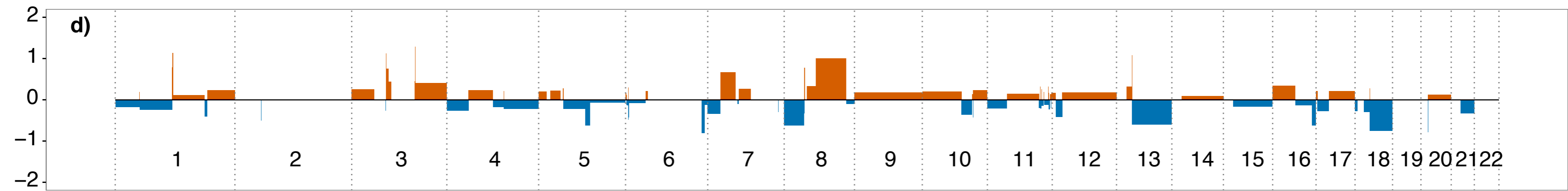

Supplement: S9 Fig — Somatic copy-number alterations detected in the cell-free DNA of ONK1. a) Plasma harvested immediately. b) Plasma harvested at 2 h. c) Plasma harvested at 4 h. d) Plasma harvested at 24 h. Y-axis; log2 of the segmented copy-number alteration ratios. X-axis; autosomal chromosomes in order. Red colour bars; amplifications. Blue colour bars; deletions. (PDF) [file pone.0168153.s015.pdf]

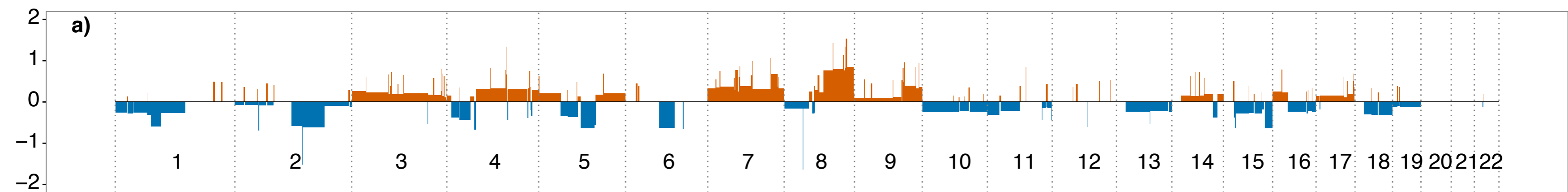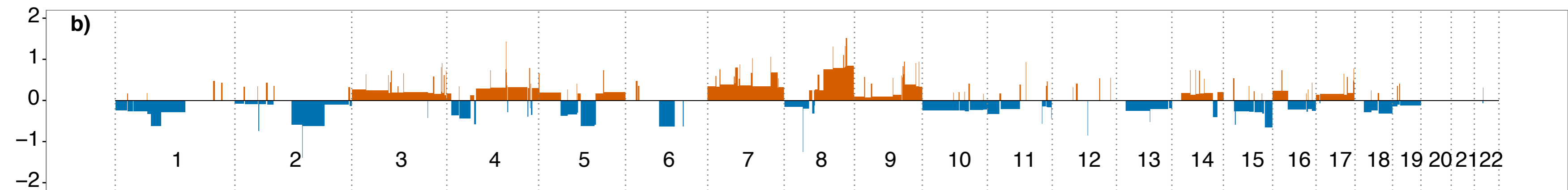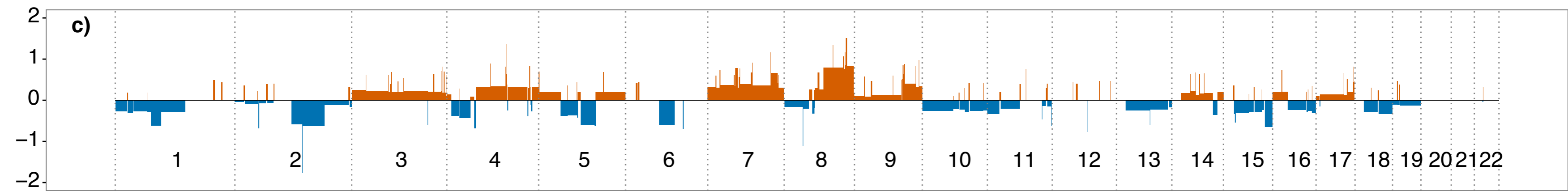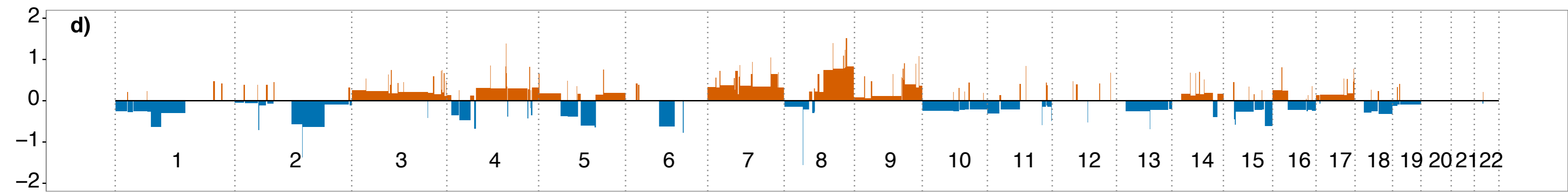

Supplement: S10 Fig — Somatic copy-number alterations detected in the cell-free DNA of ONK2. a) Plasma harvested immediately. b) Plasma harvested at 2 h. c) Plasma harvested at 4 h. d) Plasma harvested at 24 h. Y-axis; log2 of the segmented copy-number alteration ratios. X-axis; autosomal chromosomes in order. Red colour bars; amplifications. Blue colour bars; deletions. (PDF) [file pone.0168153.s016.pdf]

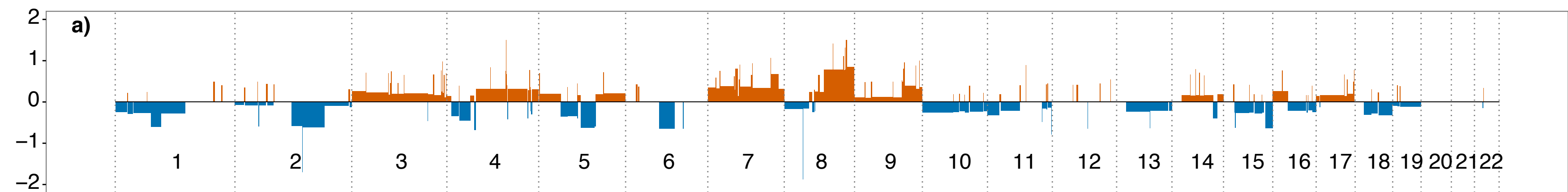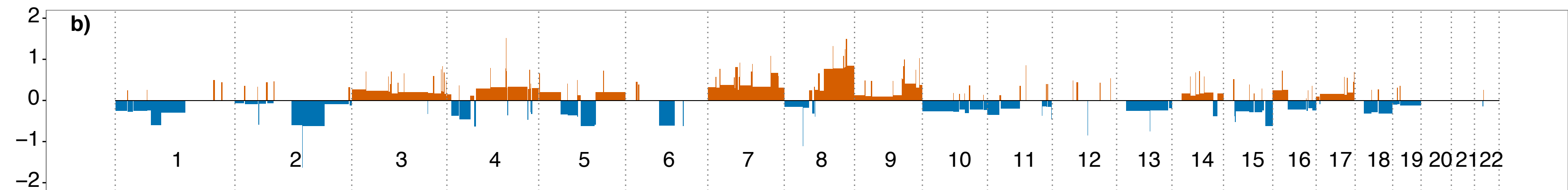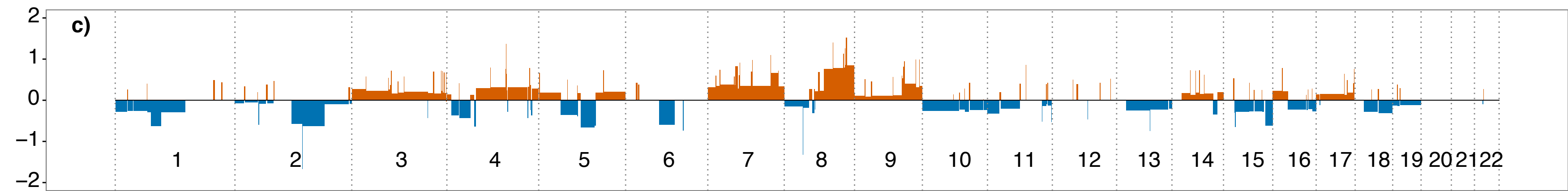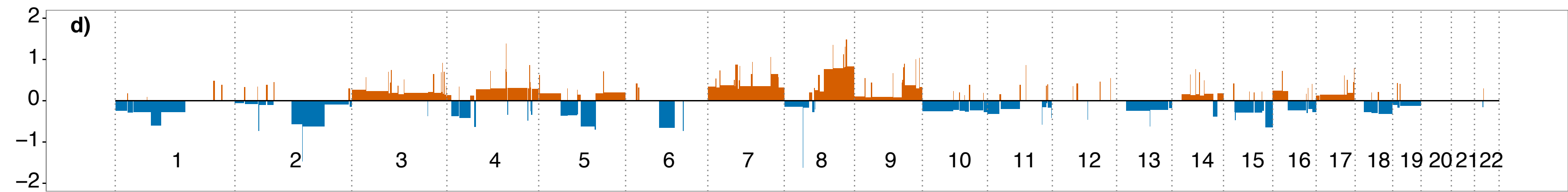

Supplement: S11 Fig — Somatic copy-number alterations detected in the cell-free DNA of ONK1. a) Plasma harvested immediately. b) Plasma harvested at 2 h. c) Plasma harvested at 4 h. d) Plasma harvested at 24 h. Y-axis; log2 of the segmented copy-number alteration ratios. X-axis; autosomal chromosomes in order. Red colour bars; amplifications. Blue colour bars; deletions. (PDF) [file pone.0168153.s017.pdf]

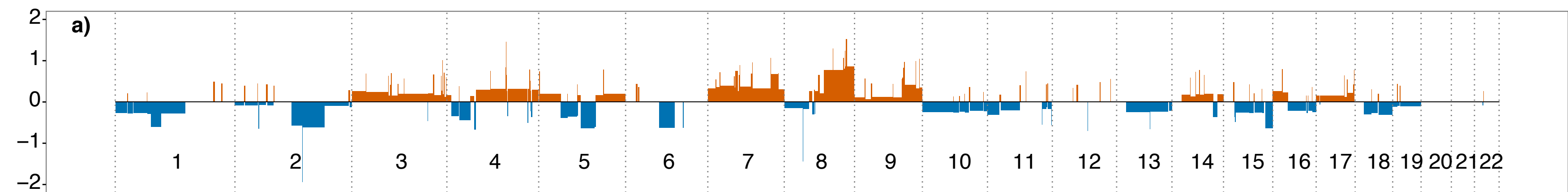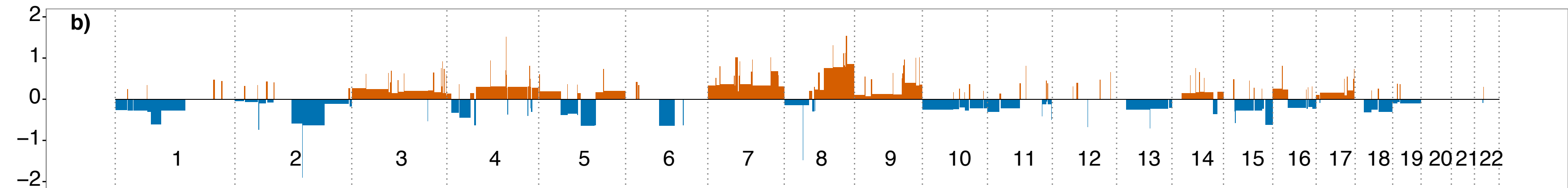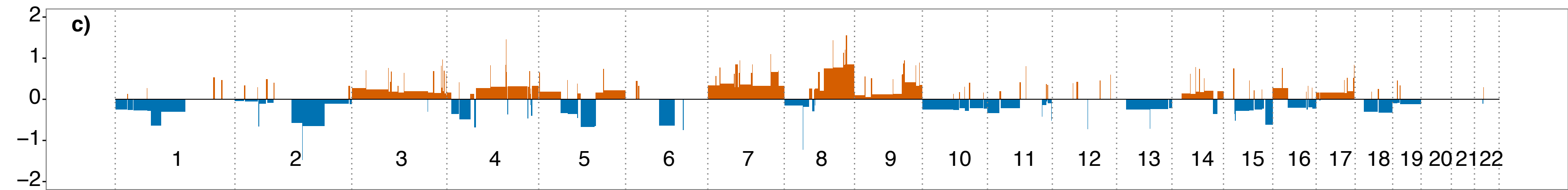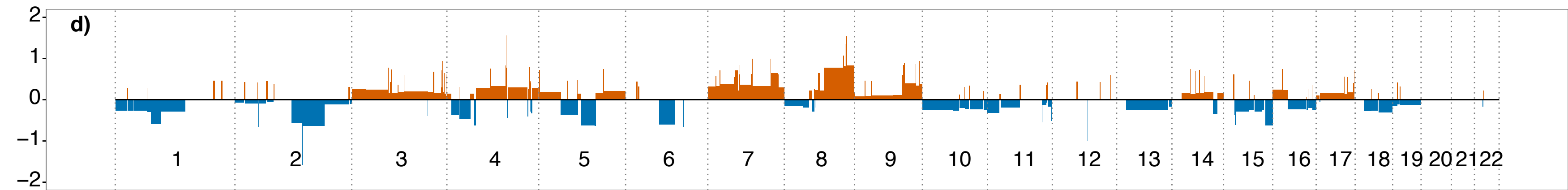

Supplement: S12 Fig — Somatic copy-number alterations detected in the cell-free DNA of ONK1. a) Plasma harvested immediately. b) Plasma harvested at 2 h. c) Plasma harvested at 4 h. d) Plasma harvested at 24 h. Y-axis; log2 of the segmented copy-number alteration ratios. X-axis; autosomal chromosomes in order. Red colour bars; amplifications. Blue colour bars; deletions. (PDF) [file pone.0168153.s018.pdf]

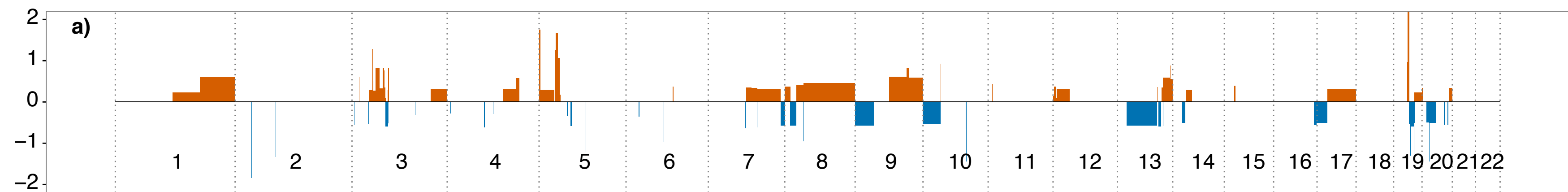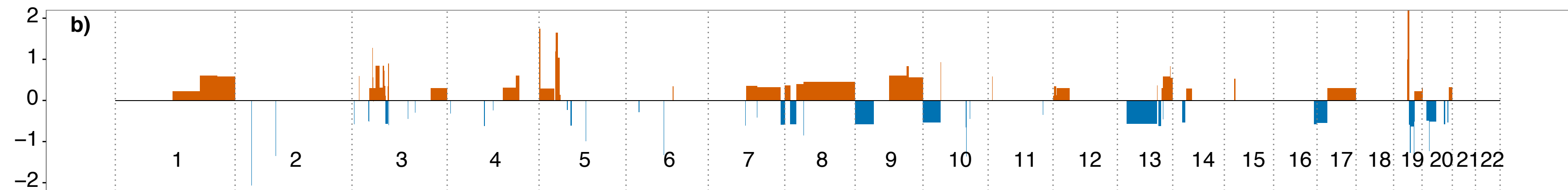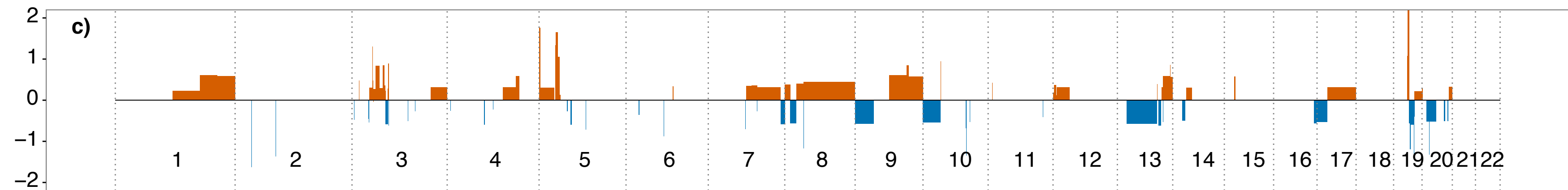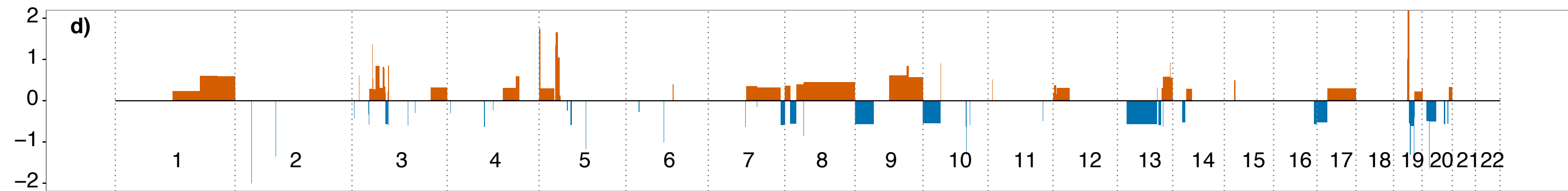

Supplement: S13 Fig — Somatic copy-number alterations detected in the cell-free DNA of ONK2. a) Plasma harvested immediately. b) Plasma harvested at 2 h. c) Plasma harvested at 6 h. d) Plasma harvested at 24 h. Y-axis; log2 of the segmented copy-number alteration ratios. X-axis; autosomal chromosomes in order. Red colour bars; amplifications. Blue colour bars; deletions. (PDF) [file pone.0168153.s019.pdf]

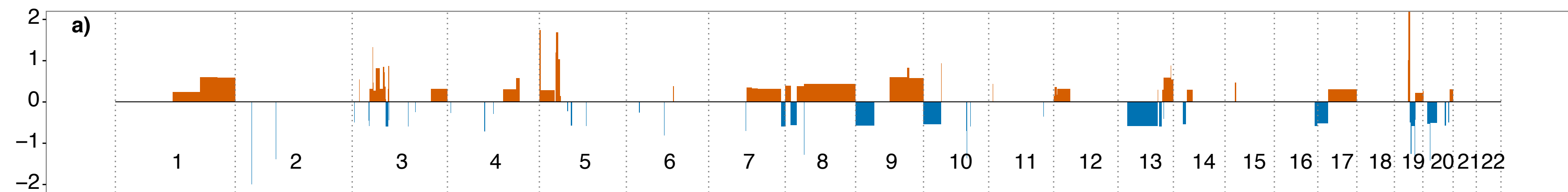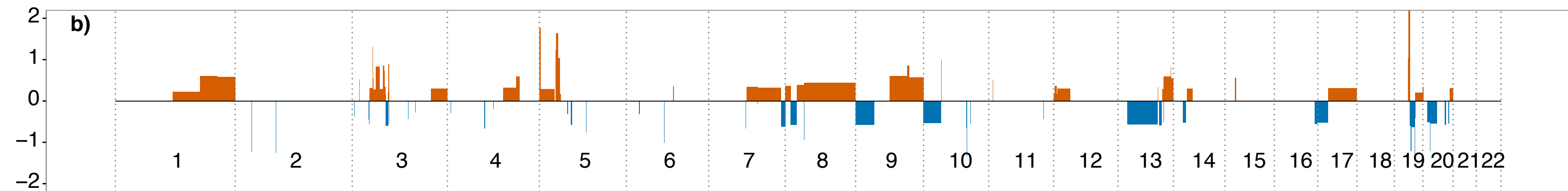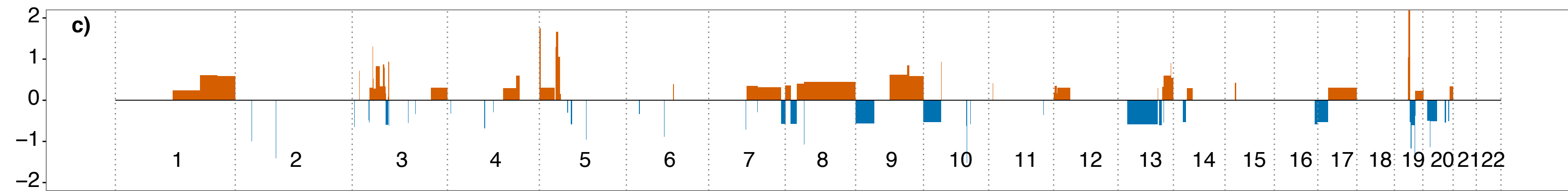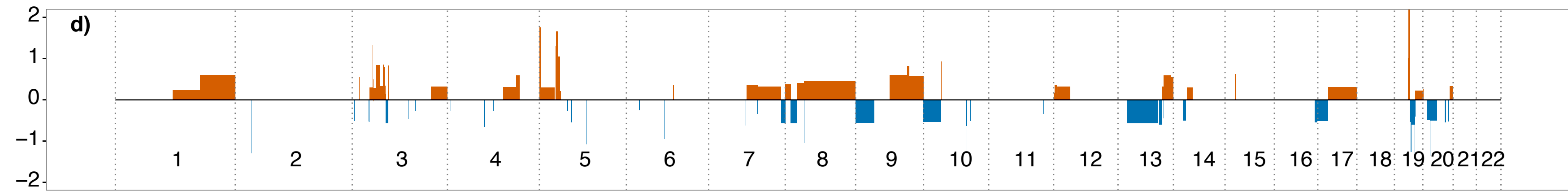

Supplement: S14 Fig — Somatic copy-number alterations detected in the cell-free DNA of ONK1. a) Plasma harvested immediately. b) Plasma harvested at 2 h. c) Plasma harvested at 6 h. d) Plasma harvested at 24 h. Y-axis; log2 of the segmented copy-number alteration ratios. X-axis; autosomal chromosomes in order. Red colour bars; amplifications. Blue colour bars; deletions. (PDF) [file pone.0168153.s020.pdf]

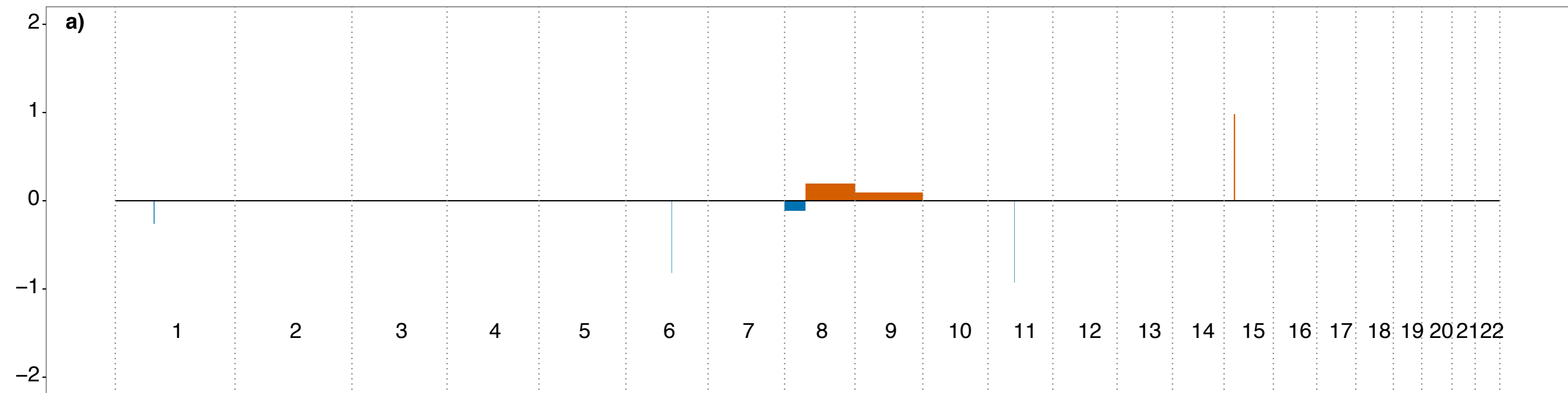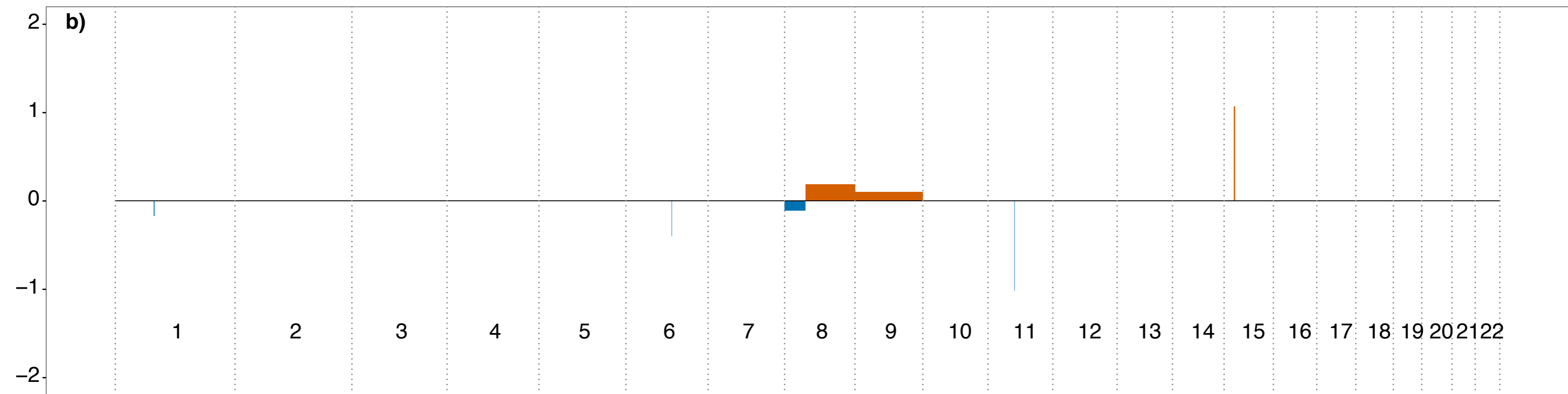

Supplement: S15 Fig — Somatic copy-number alterations detected in the cell-free DNA of ONK13. a) Plasma harvested immediately. b) Plasma harvested at 48 h. Y-axis; log2 of the segmented copy-number alteration ratios. X-axis; autosomal chromosomes in order. Red colour bars; amplifications. Blue colour bars; deletions. (PDF) [file pone.0168153.s021.pdf]

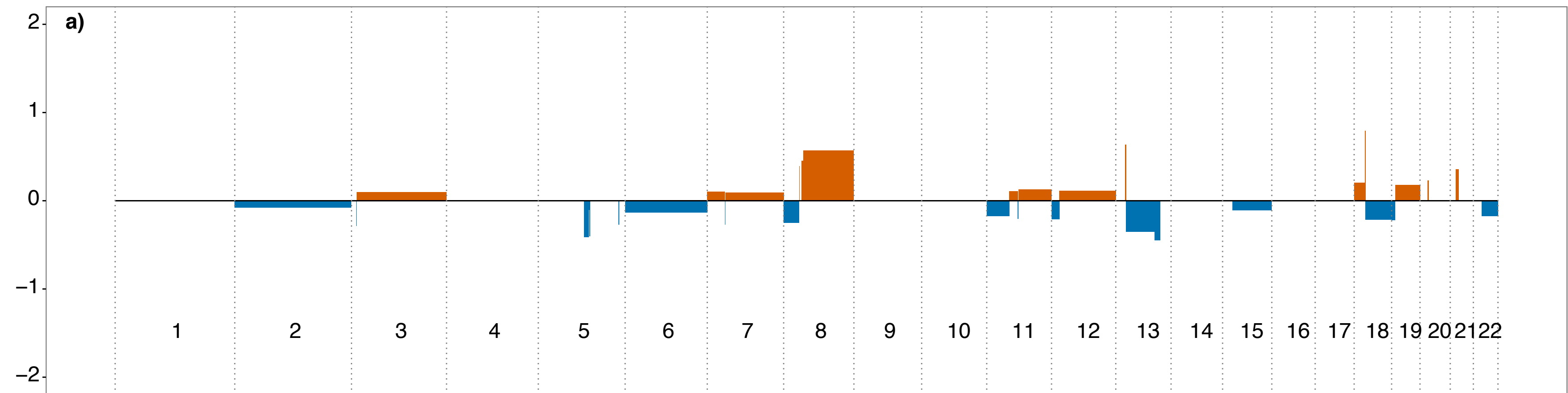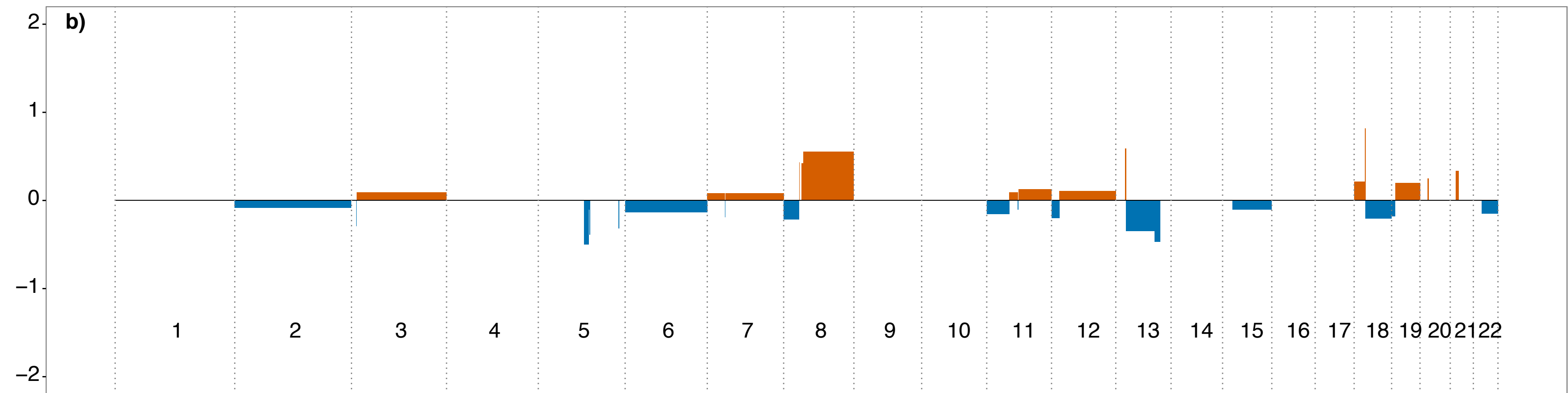

Supplement: S16 Fig — Somatic copy-number alterations detected in the cell-free DNA of ONK14. a) Plasma harvested immediately. b) Plasma harvested at 48 h. Y-axis; log2 of the segmented copy-number alteration ratios. X-axis; autosomal chromosomes in order. Red colour bars; amplifications. Blue colour bars; deletions. (PDF) [file pone.0168153.s022.pdf]

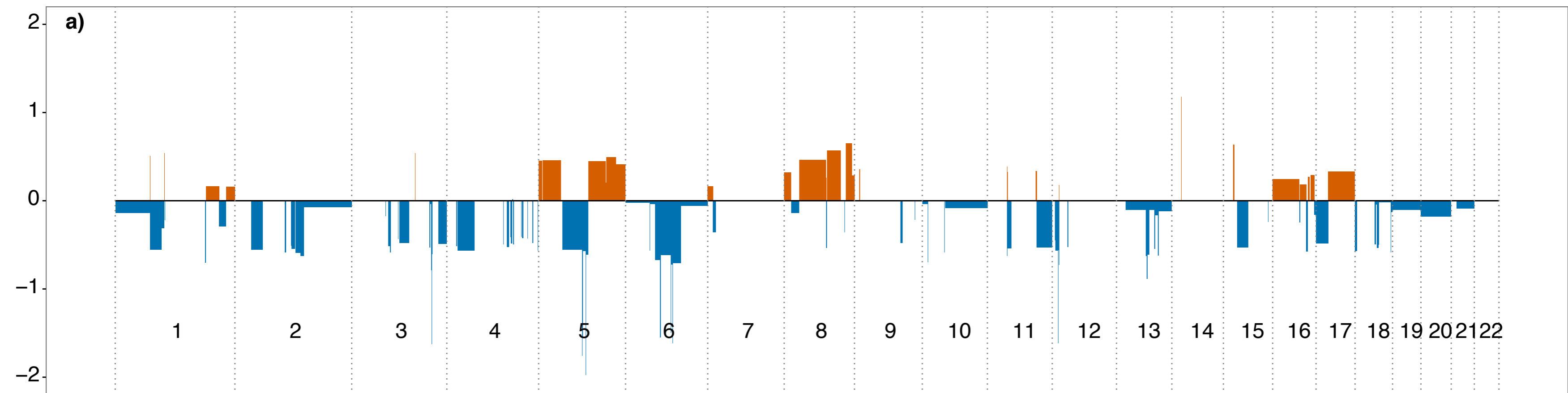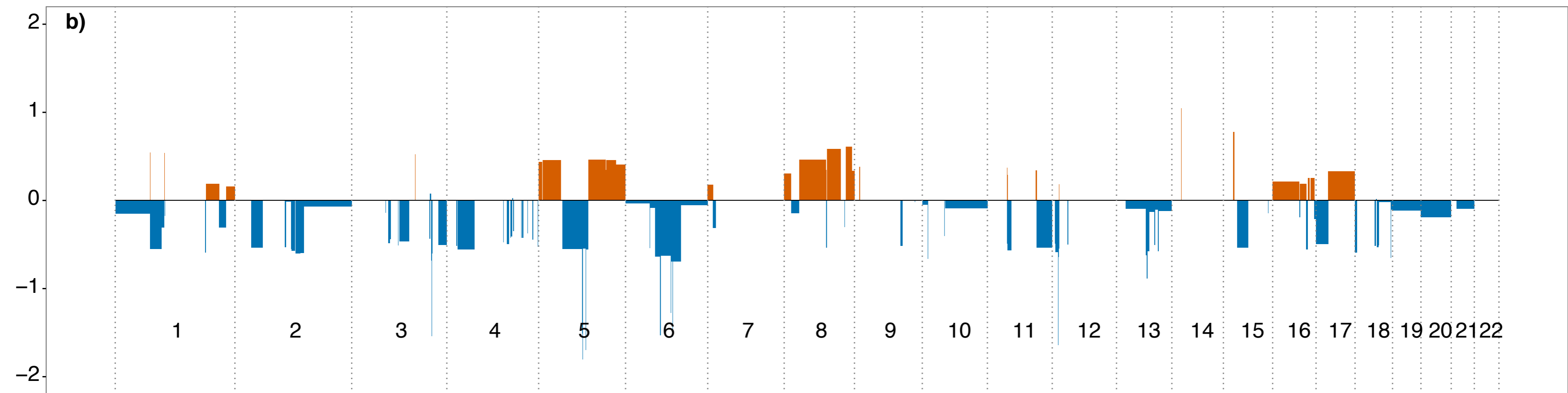

Supplement: S17 Fig — Somatic copy-number alterations detected in the cell-free DNA of ONK14. a) Plasma harvested immediately. b) Plasma harvested at 48 h. Y-axis; log2 of the segmented copy-number alteration ratios. X-axis; autosomal chromosomes in order. Red colour bars; amplifications. Blue colour bars; deletions. (PDF) [file pone.0168153.s023.pdf]

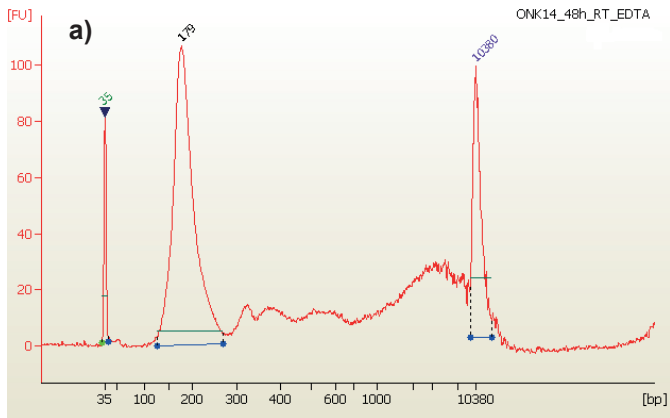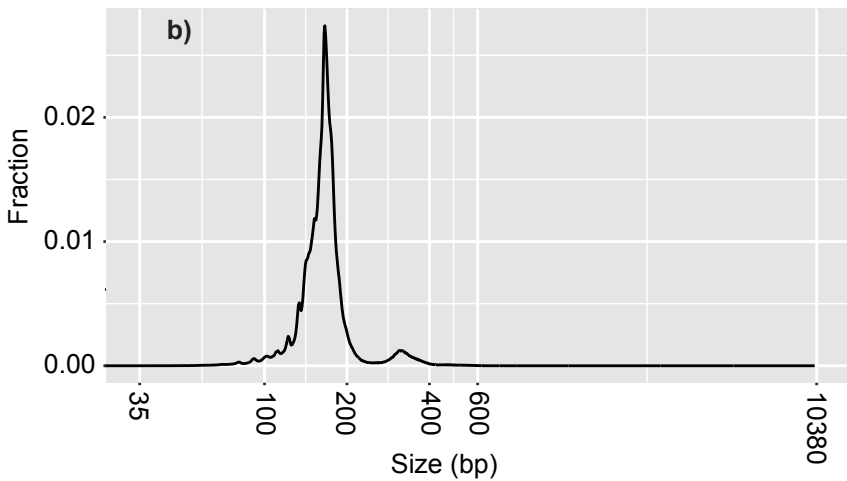

Supplement: S18 Fig — a) ONK14 plasma DNA was harvested at 48 hours and analysed on the Agilent Bioanalyzer. Large fragments >350 bp is typically not detected in high-quality cfDNA. Y-axis; arbitrary fluorescence units. X-axis; DNA fragment size in base pairs. b) The size distribution of the sequencing data. The high-molecular weight DNA is not detectable. Y-axis; fraction of all reads. X-axis; DNA fragment size in base pairs estimated from the sequencing data. (PDF) [file pone.0168153.s024.pdf]
